# Supplementary material for: Unpacking privacy: Valuation of personal data protection
Source: PLoS One. 2023 May 3;18(5):e0284581. doi: 10.1371/journal.pone.0284581 (PMC10156004; doi:10.1371/journal.pone.0284581)
Supplement: S1 Appendix — (DOCX) [file pone.0284581.s001.docx]

## Appendix 1 – Example of converting forced paired choices into a ranking

Table A1 demonstrates an example of how we convert one individual’s responses in the forced paired choice condition into a ranking. This individual was never willing to give away Mobile Phone GPS, was willing to give away Medical Records and Banking Transactions twice each and Browsing/Search/Click History and Social Media three times each. Mobile Phone GPS was ranked the most important (i.e., Willingness to Share (WTS) rank 1, importance rank 8), Medical Records and Banking Transactions was ranked second most important (i.e. WTS rank 2, importance rank 7), while Browsing/Search/Click History and Social Media will be 4th most importance (i.e., have WTS rank 4, importance rank 5).

###### Table A1. An example of ranking in Paired Choice condition.

| **Data type** | **Number of times preferred to share** | **Rank by importance** |
| --- | --- | --- |
| Banking Transactions | 2 | 7 |
| Browsing/Search/Click History | 3 | 5 |
| Electricity Use at Home | 6 | 2 |
| Loyalty Cards | 5 | 3 |
| Medical Records | 2 | 7 |
| Mobile Phone GPS | 0 | 8 |
| Physical Activity (Exercise) Tracking | 7 | 1 |
| Social Media | 3 | 5 |
