# Supplementary material for: Unpacking privacy: Valuation of personal data protection
Source: PLoS One. 2023 May 3;18(5):e0284581. doi: 10.1371/journal.pone.0284581 (PMC10156004; doi:10.1371/journal.pone.0284581)
Supplement: S2 Appendix — (DOCX) [file pone.0284581.s002.docx]

## Appendix 2 – Two randomly selected participants from the top and bottom consistency scores distribution

Rankings provided by a randomly selected participant from the bottom 5th percentile of the consistency distribution, and by a randomly selected participant in the top 5th percentile (Figure A1). Participant 49 had a low consistency score, and their responses display a lack of consistency in judging the same data types in different conditions, while Participant 149 who had a high consistency score is very consistent in their responses about data types across conditions.


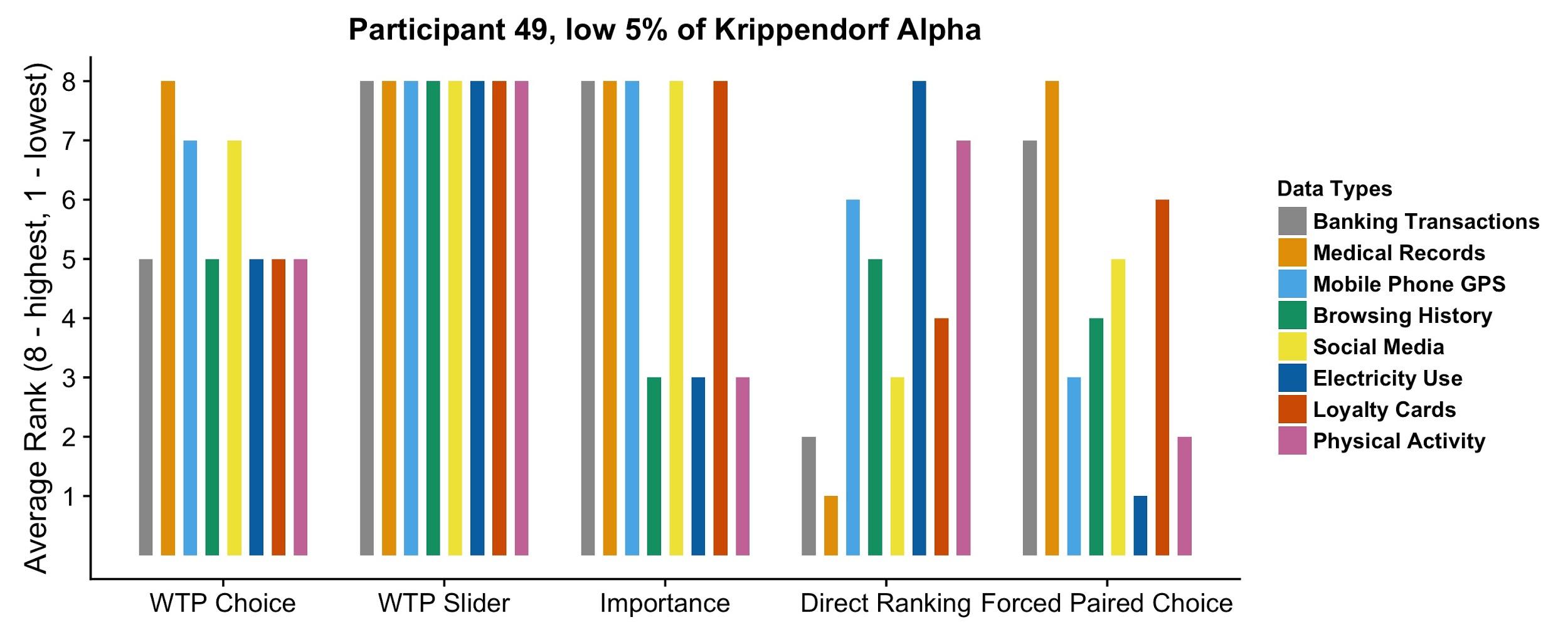


###### Figure A1a. Randomly selected participant from the bottom 5% consistency score.


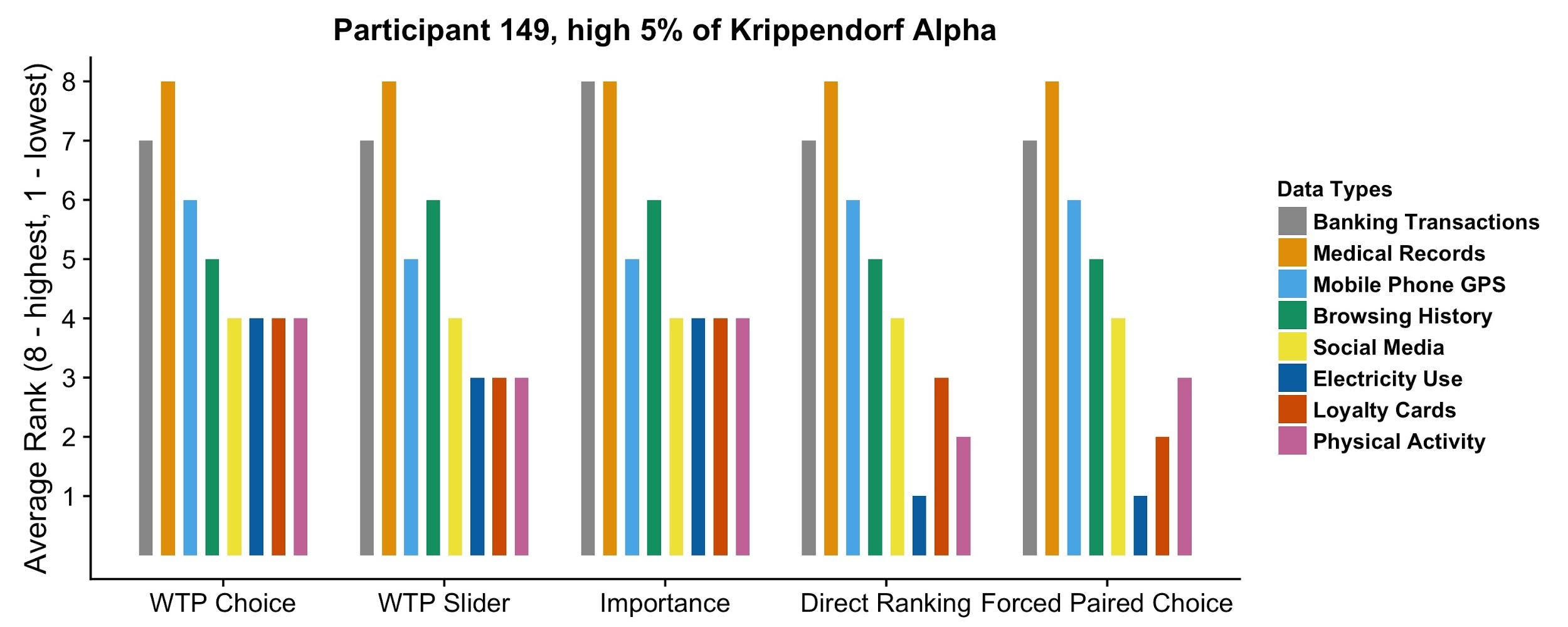


###### Figure A1b. Randomly selected participant from the top 5% consistency score.
