# Supplementary material for: Unpacking privacy: Valuation of personal data protection
Source: PLoS One. 2023 May 3;18(5):e0284581. doi: 10.1371/journal.pone.0284581 (PMC10156004; doi:10.1371/journal.pone.0284581)
Supplement: S3 Appendix — (DOCX) [file pone.0284581.s003.docx]

## Appendix 3 – tabular format of regression output

###### Table A2. Mixed-model Probit (predicting willingness to pay) and OLS (predicting the amount given people are willing to pay) for WTP Choice (Model 1 and Model 3) and WTP Slider (Model 2 and Model 4), N = 250. Banking transactions are used as a reference category. The table presents beta coefficients and 95% Confidence Intervals for each coefficient in brackets, as well as random effects, Log Likelihood. Values highlighted in bold a significant at least on p <.05 level.

|  | Probit:  Generalised linear mixed-effects | | OLS:  Linear mixed-effects | |
| --- | --- | --- | --- | --- |
|  | Model 1 | Model 2 | Model 3 | Model 4 |
| Intercept | **1.72**  **[1.38; 2.07]** | **2.38**  **[1.88; 2.87]** | **28.09**  **[22.40; 33.78]** | **22.76**  **[17.77; 27.7]** |
| Medical Records | **-0.93**  **[-1.25; -0.61]** | **-0.87**  **[-1.28; -0.46]** | -3.10  [-7.36; 1.17] | -0.27  [-3.08; 2.55] |
| Mobile Phone GPS | 0.02  [-0.34; 0.38] | **-1.91**  **[-2.22; 1.40]** | 0.32  [-3.95; 4.58] | **-10.98**  **[-13.79; -8.17]** |
| Browsing History | **-1.90**  **[-2.23; -1.58]** | **-1.74**  **[-2.15; -1.34]** | **-15.97**  **[-20.23; -11.70]** | **-10.25**  **[-13.07; -7.44]** |
| Social Media | **-1.99**  **[-2.31; -1.66]** | **-1.78**  **[2.18; -1.37]** | **-18.05**  **[-22.31; -13.79]** | **-11.36**  **[-14.17; -8.55]** |
| Electricity Use | **-3.21**  **[-3.59; -2.83]** | **-3.05**  **[-3.48; -2.63]** | **-21.54**  **[-25.81; -17.28]** | **-15.54**  **[-18.35; -12.72]** |
| Loyalty Cards | **-3.37**  **[-3.48; -2.73]** | **-2.96**  **[-3.39; -2.54]** | **-21.57**  **[-25.83; -17.30]** | **-15.07**  **[-17.88; -12.25]** |
| Physical Activity | **-3.37**  **[-3.76; -2.97]** | **-3.25**  **[-3.69; -2.81]** | **-22.20**  **[-26.46; -17.93]** | **-15.64**  **[-18.46; -12.83]** |
| Standardised Consistency | **0.43**  **[0.32; 0.55]** | **0.18**  **[0.01; 0.35]** | 1.94  [-0.42; 4.30] | -1.38  [-3.60; 0.83] |
| Standardised Age | 0.08  [-0.03; 0.20] | -**0.29**  **[-0.46; -0.12]** | -0.95  [-3.32; 1.42] | **-2.27**  **[-4.50; -0.05]** |
| Female | -0.20  [-0.43; 0.03] | -0.12  [-0.48; 0.23] | **-8.06**  **[-13.11; -3.02]** | **-5.13**  **[-9.86; -0.38]** |
| Degree: Undergraduate | 0.06  [-0.18; 0.30] | 0.02  [-0.34; 0.38] | -3.12  [-8.27; 2.03] | -1.85  [-6.69; 2.98] |
| Degree: Postgraduate | **0.48**  **[0.12; 0.84]** | 0.31  [-0.25; 0.88] | **18.61**  **[10.84; 26.39]** | **10.06**  **[2.76; 17.35]** |
| Standardised income | -0.08  [-0.19; 0.03] | -0.08  [-0.25; 0.09] | -1.22  [-3.61; 1.17] | -1.28  [-3.53; 0.96] |
| Random effects | | | | |
| Intercept σ (participant) | 0.38 | 1.34 | 273.0 | 273.3 |
| Model Fit | | | | |
| Deviance (-2LL) | -811.97 | -936.93 | 18,894.57 | 17,413.04 |
