# Supplementary material for: Unpacking privacy: Valuation of personal data protection
Source: PLoS One. 2023 May 3;18(5):e0284581. doi: 10.1371/journal.pone.0284581 (PMC10156004; doi:10.1371/journal.pone.0284581)
Supplement: S1 File — (DOCX) [file pone.0284581.s004.docx]

# **Supplementary Materials for *Willingness to Pay to Protect Personal Data***

Experiment - Value Personal Data - June 2017

Survey Flow

Block: Consent (1 Question)

Branch: New Branch

If

If Welcome to this study on the value of personal data   About the study The aim of the study is to... I have read the information above Is Not Selected

Or Welcome to this study on the value of personal data   About the study The aim of the study is to... I have received enough information about the study and the intended uses of any data I supply Is Not Selected

Or Welcome to this study on the value of personal data   About the study The aim of the study is to... I understand that I am free to stop the study at any time Is Not Selected

Or Welcome to this study on the value of personal data   About the study The aim of the study is to... I consent to take part in the study Is Not Selected

EndSurvey: Advanced

Standard: Demographics (5 Questions)

Standard: About the App (1 Question)

BlockRandomizer: 5 - Evenly Present Elements

Branch: New Branch

If

If Please enter your full Prolific ID Text Response Is Displayed

BlockRandomizer: 8 - Evenly Present Elements

Standard: CondA_Browsing (19 Questions)

Standard: CondA_MedRec (19 Questions)

Standard: CondA_Loyalty Cards (19 Questions)

Standard: CondA_Physical (19 Questions)

Standard: CondA_Social (19 Questions)

Standard: CondA_Electricity (19 Questions)

Standard: CondA_Mobile GPS (19 Questions)

Standard: CondA_Banking (19 Questions)

Standard: Comments A (1 Question)

Branch: New Branch

If

If Please enter your full Prolific ID Text Response Is Displayed

Standard: Condition B (L&M version) (1 Question)

Standard: Comments B (1 Question)

Branch: New Branch

If

If Please enter your full Prolific ID Text Response Is Displayed

Standard: Condition C (2 Questions)

Standard: Comments C (1 Question)

Standard: Condition D (2 Questions)

Branch: New Branch

If

If Please enter your full Prolific ID Text Response Is Displayed

Standard: Condition E (1 Question)

Standard: Comments E (1 Question)

Standard: Good bye (1 Question)

| Page Break |  |
| --- | --- |

Start of Block: Consent

Intro and Consent Welcome to this study on the value of personal data
 
About the study
The aim of the study is to understand whether people value their personal, digitally recorded data and if so, how much. You will be shown hypothetical scenarios and asked what you would choose to do in those scenarios. These scenarios will be about different types of personal data. There are no right or wrong answers, and your honest responses are important to us. The study is expected to take 30 minutes and you will receive £3.

 Confidentiality
All information obtained during the study will be kept confidential. You data may be published in academic journals and presented at conferences. Whether published or not, the information will not be identified as yours.

 Stopping the study
You can stop the study at any time. If you stop before the end, your data will not be used. However, if you reach the end of the study we will not be able to remove your data because we will not be able to identify the data as yours. If you stop before the end you will forfeit your payment but there will be no other consequences.
 
Risks and benefits
There are no special risks associated with this study, beyond normal risks associated with computer use. The benefits of the study will be to improve our understanding of the value of online data protection. The study has been approved by the HSSREC committee at the University of Warwick.

 Complaints procedure
Any complaint about the way you have been treated during the study or any possible harm you may have suffered will be addressed. You can send your complaint to the person below, who is a senior official of the University of Warwick and is independent of this research.
 
Head of Research Governance
 Research & Impact Services
 University House
 University of Warwick
 Coventry
 CV4 8UW Email: researchgovernance@warwick.ac.uk Tel: 024 76 522746

 If you would like to take part in the study, please state your consent by confirming the following:

- I have read the information above (1)
- I have received enough information about the study and the intended uses of any data I supply (2)
- I understand that I am free to stop the study at any time (3)
- I consent to take part in the study (4)

End of Block: Consent

Start of Block: Demographics

Prol_ID Please enter your full Prolific ID

________________________________________________________________

| Page Break |  |
| --- | --- |

| 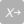 |
| --- |

YearBorn What year were you born?

▼ I'd prefer not to answer (1) ... 1900 (102)

| 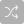 |
| --- |

Gender What is your gender?

- Male (1)
- Female (2)
- Other (please describe if you wish) (3) ________________________________________________
- I'd prefer not to answer (4)

| 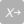 |
| --- |

Education What is the highest level of education you have completed?

- Primary school or less (1)
- Secondary school (e.g. GCSEs, O Levels) (2)
- Sixth form or college (e.g. A Levels, IB) (3)
- Undergraduate degree or equivalent (4)
- Masters degree or equivalent (5)
- PhD or equivalent (6)
- Other (please describe if you wish) (7) ________________________________________________
- I'd prefer not to answer (8)

| 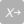 |
| --- |

Income What is your household's annual income before tax?

- less than £10,000 (1)
- £10,000 to £19,999 (2)
- £20,000 to £29,999 (3)
- £30,000 to £39,999 (4)
- £40,000 to £49,999 (5)
- £50,000 to £74,999 (6)
- £75,000 to £99,999 (7)
- £100,000 to £149,999 (8)
- £150,000 to £199,999 (9)
- £200,000 or more (10)
- I'd prefer not to answer (11)

End of Block: Demographics

Start of Block: About the App

AboutApp
**Please read the instructions for the study.**
  Imagine you have just bought a new smartphone. In addition to having all the usual functions (camera, phone, Internet) it also has a special application that aims to assist you in your everyday life by tracking items of personal data. This personal data could include various aspects of your day and any transactions you make (including where you’ve been via GPS data, your bank records, your household bills, your social networking activities etc). Using this app will make your everyday life easier by saving you time in organizing and planning your expenses and schedule.

 However, using the app requires you to share some pieces of your data with the app providers. The app providers can either collect information about you when you use the app to use it for various marketing and other purposes, or they can keep your data private. In this case your data won’t be linked to your identity directly but placed along with other people’s data in their database and only used to maintain the functionality of your app.

 During this study we will ask you a number of questions about personal data. You will be required to make decisions based on how valuable and/or important various pieces of personal data are to you. The decisions are hypothetical but please answer them as if they were real.

End of Block: About the App

Start of Block: CondA_Browsing

CondA_Brow_Intro **${lm://Field/1}**
When you first use the personal data app you are given two choices; either you can run the app in a “free” mode or or you can use it in “paid” mode. In “free” mode, you do not have to pay anything but the company providing the app will collect information about you when you use it. In “paid” mode, you pay money for the service and your data will remain private. If you choose this option, your data will not be used by the company providing the service for any purposes apart from maintaining the functionality of the app.

| 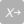 |
| --- |

CondA_Brow_Any
Would you be willing to pay something, even a very small amount, to keep the **${lm://Field/1}** data private, or would you use the app for free and share the data with the app provider?

- I would pay something to keep the data private. (1)
- I would use the app for free and share the data. (2)

Skip To: CondA_Brow_40 If Would you be willing to pay something, even a very small amount, to keep the ${lm://Field/1} data... = I would pay something to keep the data private.

Skip To: End of Block If Would you be willing to pay something, even a very small amount, to keep the ${lm://Field/1} data... = I would use the app for free and share the data.

| 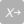 |
| --- |

CondA_Brow_40
**${lm://Field/1}**

Would you be willing to pay £40 per month to keep the **${lm://Field/1}**data private?

- I would pay £40 to keep the data private. (1)
- I would not pay £40 to keep the data private. (2)

Skip To: CondA_Brow_125 If ${lm://Field/1} Would you be willing to pay £40 per month to keep the ${lm://Field/1} data privat... = I would pay £40 to keep the data private.

Skip To: CondA_Brow_15 If ${lm://Field/1} Would you be willing to pay £40 per month to keep the ${lm://Field/1} data privat... = I would not pay £40 to keep the data private.

CondA_Brow_125
**${lm://Field/1}**

Would you be willing to pay £125 per month to keep the **${lm://Field/1}** data private?

- I would pay £125 to keep the data private. (1)
- I would not pay £125 to keep the data private. (2)

Skip To: CondA_Brow_175 If ${lm://Field/1} Would you be willing to pay £125 per month to keep the ${lm://Field/1} data priva... = I would pay £125 to keep the data private.

Skip To: CondA_Brow_75 If ${lm://Field/1} Would you be willing to pay £125 per month to keep the ${lm://Field/1} data priva... = I would not pay £125 to keep the data private.

| 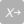 |
| --- |

CondA_Brow_15
**${lm://Field/1}**

Would you be willing to pay £15 per month to keep the **${lm://Field/1}** data private?

- I would pay £15 to keep the data private. (1)
- I would not pay £15 to keep the data private. (2)

Skip To: CondA_Brow_25 If ${lm://Field/1} Would you be willing to pay £15 per month to keep the ${lm://Field/1} data privat... = I would pay £15 to keep the data private.

Skip To: CondA_Brow_5 If ${lm://Field/1} Would you be willing to pay £15 per month to keep the ${lm://Field/1} data privat... = I would not pay £15 to keep the data private.

CondA_Brow_175
**${lm://Field/1}**

Would you be willing to pay £175 per month to keep the **${lm://Field/1}** data private?

- I would pay £175 to keep the data private (1)
- I would not pay £175 to keep the data private (2)

Skip To: CondA_Brow_200 If ${lm://Field/1} Would you be willing to pay £175 per month to keep the ${lm://Field/1} data priva... = I would pay £175 to keep the data private

Skip To: CondA_Brow_150 If ${lm://Field/1} Would you be willing to pay £175 per month to keep the ${lm://Field/1} data priva... = I would not pay £175 to keep the data private

CondA_Brow_75
**${lm://Field/1}**

Would you be willing to pay £75 per month to keep the **${lm://Field/1}** data private?

- I would pay £75 to keep the data private (1)
- I would not pay £75 to keep the data private (2)

Skip To: CondA_Brow_100 If ${lm://Field/1} Would you be willing to pay £75 per month to keep the ${lm://Field/1} data privat... = I would pay £75 to keep the data private

Skip To: CondA_Brow_50 If ${lm://Field/1} Would you be willing to pay £75 per month to keep the ${lm://Field/1} data privat... = I would not pay £75 to keep the data private

CondA_Brow_25
**${lm://Field/1}**

Would you be willing to pay £25 per month to keep the **${lm://Field/1}** data private?

- I would pay £25 to keep the data private (1)
- I would not pay £25 to keep the data private (2)

Skip To: CondA_Brow_30 If ${lm://Field/1} Would you be willing to pay £25 per month to keep the ${lm://Field/1} data privat... = I would pay £25 to keep the data private

Skip To: CondA_Brow_20 If ${lm://Field/1} Would you be willing to pay £25 per month to keep the ${lm://Field/1} data privat... = I would not pay £25 to keep the data private

CondA_Brow_5
**${lm://Field/1}**

Would you be willing to pay £5 per month to keep the **${lm://Field/1}** data private?

- I would pay £5 to keep the data private (1)
- I would not pay £5 to keep the data private (2)

Skip To: CondA_Brow_10 If ${lm://Field/1} Would you be willing to pay £5 per month to keep the ${lm://Field/1} data private... = I would pay £5 to keep the data private

Skip To: CondA_Brow_1pound If ${lm://Field/1} Would you be willing to pay £5 per month to keep the ${lm://Field/1} data private... = I would not pay £5 to keep the data private

CondA_Brow_200
**${lm://Field/1}**

Would you be willing to pay £200 per month to keep the **${lm://Field/1}** data private?

- I would pay £200 to keep the data private (1)
- I would not pay £200 to keep the data private (2)

Skip To: CondA_Brow_Max If ${lm://Field/1} Would you be willing to pay £200 per month to keep the ${lm://Field/1} data priva... = I would pay £200 to keep the data private

Skip To: End of Block If ${lm://Field/1} Would you be willing to pay £200 per month to keep the ${lm://Field/1} data priva... = I would not pay £200 to keep the data private

CondA_Brow_150
**${lm://Field/1}**

Would you be willing to pay £150 per month to keep the **${lm://Field/1}** data private?

- I would pay £150 to keep the data private (1)
- I would not pay £150 to keep the data private (2)

Skip To: End of Block If ${lm://Field/1} Would you be willing to pay £150 per month to keep the ${lm://Field/1} data priva... = I would pay £150 to keep the data private

Skip To: End of Block If ${lm://Field/1} Would you be willing to pay £150 per month to keep the ${lm://Field/1} data priva... = I would not pay £150 to keep the data private

CondA_Brow_100
**${lm://Field/1}**

Would you be willing to pay £100 per month to keep the **${lm://Field/1}** data private?

- I would pay £100 to keep the data private (1)
- I would not pay £100 to keep the data private (2)

Skip To: End of Block If ${lm://Field/1} Would you be willing to pay £100 per month to keep the ${lm://Field/1} data priva... = I would pay £100 to keep the data private

Skip To: End of Block If ${lm://Field/1} Would you be willing to pay £100 per month to keep the ${lm://Field/1} data priva... = I would not pay £100 to keep the data private

CondA_Brow_50
**${lm://Field/1}**

Would you be willing to pay £50 per month to keep the **${lm://Field/1}** data private?

- I would pay £50 to keep the data private (1)
- I would not pay £50 to keep the data private (2)

Skip To: End of Block If ${lm://Field/1} Would you be willing to pay £50 per month to keep the ${lm://Field/1} data privat... = I would pay £50 to keep the data private

Skip To: End of Block If ${lm://Field/1} Would you be willing to pay £50 per month to keep the ${lm://Field/1} data privat... = I would not pay £50 to keep the data private

CondA_Brow_30
**${lm://Field/1}**

Would you be willing to pay £30 per month to keep the **${lm://Field/1}** data private?

- I would pay £30 to keep the data private (1)
- I would not pay £30 to keep the data private (2)

Skip To: End of Block If ${lm://Field/1} Would you be willing to pay £30 per month to keep the ${lm://Field/1} data privat... = I would pay £30 to keep the data private

Skip To: End of Block If ${lm://Field/1} Would you be willing to pay £30 per month to keep the ${lm://Field/1} data privat... = I would not pay £30 to keep the data private

CondA_Brow_20
**${lm://Field/1}**

Would you be willing to pay £20 per month to keep the **${lm://Field/1}** data private, or would you use the app for free and share the data?

- I would pay £20 to keep the data private (1)
- I would not pay £20 to keep the data private (2)

Skip To: End of Block If ${lm://Field/1} Would you be willing to pay £20 per month to keep the ${lm://Field/1} data privat... = I would pay £20 to keep the data private

Skip To: End of Block If ${lm://Field/1} Would you be willing to pay £20 per month to keep the ${lm://Field/1} data privat... = I would pay £20 to keep the data private

CondA_Brow_10
**${lm://Field/1}**

Would you be willing to pay £10 per month to keep the **${lm://Field/1}** data private?

- I would pay £10 to keep the data private (1)
- I would not pay £10 to keep the data private (2)

Skip To: End of Block If ${lm://Field/1} Would you be willing to pay £10 per month to keep the ${lm://Field/1} data private? = I would pay £10 to keep the data private

Skip To: End of Block If ${lm://Field/1} Would you be willing to pay £10 per month to keep the ${lm://Field/1} data private? = I would not pay £10 to keep the data private

| 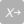 |
| --- |

CondA_Brow_1pound
**${lm://Field/1}**

Would you be willing to pay £1 per month to keep the **${lm://Field/1}** data private?

- I would pay £1 to keep the data private (1)
- I would not pay £1 to keep the data private (2)

Skip To: End of Block If ${lm://Field/1} Would you be willing to pay £1 per month to keep the ${lm://Field/1} data private... = I would pay £1 to keep the data private

Skip To: CondA_Brow_1penny If ${lm://Field/1} Would you be willing to pay £1 per month to keep the ${lm://Field/1} data private... = I would not pay £1 to keep the data private

| 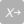 |
| --- |

CondA_Brow_1penny
**${lm://Field/1}**

Would you be willing to pay 1p per month to keep the **${lm://Field/1}** data private?

- I would pay 1p to keep the data private (1)
- I would not pay 1p to keep the data private (2)

Skip To: End of Block If ${lm://Field/1} Would you be willing to pay 1p per month to keep the ${lm://Field/1} data private?  = I would pay 1p to keep the data private

Skip To: End of Block If ${lm://Field/1} Would you be willing to pay 1p per month to keep the ${lm://Field/1} data private?  = I would not pay 1p to keep the data private

CondA_Brow_Max
**${lm://Field/1}**

You said you would be willing to pay £200 per month to keep the **${lm://Field/1}** data private. What is the maximum amount of money you would be willing to pay per month to keep this data private?

- Each month, I would pay up to £ (1) ________________________________________________

End of Block: CondA_Browsing

Start of Block: CondA_MedRec

CondA_MedRec_intro **${lm://Field/1}**
When you first use the personal data app you are given two choices; either you can run the app in a “free” mode or or you can use it in “paid” mode. In “free” mode, you do not have to pay anything but the company providing the app will collect information about you when you use it. In “paid” mode, you pay money for the service and your data will remain private. If you choose this option, your data will not be used by the company providing the service for any purposes apart from maintaining the functionality of the app.

CondA_MedRec_any
Would you be willing to pay something, even a very small amount, to keep the **${lm://Field/1}** data private, or would you use the app for free and share the data with the app provider?

- I would pay something to keep the data private. (1)
- I would use the app for free and share the data. (2)

Skip To: CondA_MedRec_40 If Would you be willing to pay something, even a very small amount, to keep the ${lm://Field/1} data... = I would pay something to keep the data private.

Skip To: End of Block If Would you be willing to pay something, even a very small amount, to keep the ${lm://Field/1} data... = I would use the app for free and share the data.

CondA_MedRec_40
${lm://Field/1}

Would you be willing to pay £40 per month to keep the **${lm://Field/1}** data private?

- I would pay £40 to keep the data private. (1)
- I would not pay £40 to keep the data private. (2)

Skip To: CondA_MedRec_125 If ${lm://Field/1} Would you be willing to pay £40 per month to keep the ${lm://Field/1} data privat... = I would pay £40 to keep the data private.

Skip To: CondA_MedRec_15 If ${lm://Field/1} Would you be willing to pay £40 per month to keep the ${lm://Field/1} data privat... = I would not pay £40 to keep the data private.

CondA_MedRec_125
${lm://Field/1}

Would you be willing to pay £125 per month to keep the **${lm://Field/1}** data private?

- I would pay £125 to keep the data private. (1)
- I would not pay £125 to keep the data private. (2)

Skip To: CondA_MedRec_175 If ${lm://Field/1} Would you be willing to pay £125 per month to keep the ${lm://Field/1} data priva... = I would pay £125 to keep the data private.

Skip To: CondA_MedRec_75 If ${lm://Field/1} Would you be willing to pay £125 per month to keep the ${lm://Field/1} data priva... = I would not pay £125 to keep the data private.

CondA_MedRec_15
${lm://Field/1}

Would you be willing to pay £15 per month to keep the **${lm://Field/1}** data private?

- I would pay £15 to keep the data private. (1)
- I would not pay £15 to keep the data private. (2)

Skip To: CondA_MedRec_25 If ${lm://Field/1} Would you be willing to pay £15 per month to keep the ${lm://Field/1} data privat... = I would pay £15 to keep the data private.

Skip To: CondA_MedRec_5 If ${lm://Field/1} Would you be willing to pay £15 per month to keep the ${lm://Field/1} data privat... = I would not pay £15 to keep the data private.

CondA_MedRec_175
${lm://Field/1}

Would you be willing to pay £175 per month to keep the **${lm://Field/1}** data private?

- I would pay £175 to keep the data private (1)
- I would not pay £175 to keep the data private (2)

Skip To: CondA_MedRec_200 If ${lm://Field/1} Would you be willing to pay £175 per month to keep the ${lm://Field/1} data priva... = I would pay £175 to keep the data private

Skip To: CondA_MedRec_150 If ${lm://Field/1} Would you be willing to pay £175 per month to keep the ${lm://Field/1} data priva... = I would not pay £175 to keep the data private

CondA_MedRec_75
${lm://Field/1}

Would you be willing to pay £75 per month to keep the **${lm://Field/1}** data private?

- I would pay £75 to keep the data private (1)
- I would not pay £75 to keep the data private (2)

Skip To: CondA_MedRec_100 If ${lm://Field/1} Would you be willing to pay £75 per month to keep the ${lm://Field/1} data privat... = I would pay £75 to keep the data private

Skip To: CondA_MedRec_50 If ${lm://Field/1} Would you be willing to pay £75 per month to keep the ${lm://Field/1} data privat... = I would not pay £75 to keep the data private

CondA_MedRec_25
${lm://Field/1}

Would you be willing to pay £25 per month to keep the **${lm://Field/1}** data private?

- I would pay £25 to keep the data private (1)
- I would not pay £25 to keep the data private (2)

Skip To: CondA_MedRec_30 If ${lm://Field/1} Would you be willing to pay £25 per month to keep the ${lm://Field/1} data privat... = I would pay £25 to keep the data private

Skip To: CondA_MedRec_20 If ${lm://Field/1} Would you be willing to pay £25 per month to keep the ${lm://Field/1} data privat... = I would not pay £25 to keep the data private

CondA_MedRec_5
${lm://Field/1}

Would you be willing to pay £5 per month to keep the **${lm://Field/1}** data private?

- I would pay £5 to keep the data private (1)
- I would not pay £5 to keep the data private (2)

Skip To: CondA_MedRec_10 If ${lm://Field/1} Would you be willing to pay £5 per month to keep the ${lm://Field/1} data private... = I would pay £5 to keep the data private

Skip To: CondA_MedRec_1pound If ${lm://Field/1} Would you be willing to pay £5 per month to keep the ${lm://Field/1} data private... = I would not pay £5 to keep the data private

CondA_MedRec_200
${lm://Field/1}

Would you be willing to pay £200 per month to keep the **${lm://Field/1}** data private?

- I would pay £200 to keep the data private (1)
- I would not pay £200 to keep the data private (2)

Skip To: CondA_MedRec_MAX If ${lm://Field/1} Would you be willing to pay £200 per month to keep the ${lm://Field/1} data priva... = I would pay £200 to keep the data private

Skip To: End of Block If ${lm://Field/1} Would you be willing to pay £200 per month to keep the ${lm://Field/1} data priva... = I would not pay £200 to keep the data private

CondA_MedRec_150
${lm://Field/1}

Would you be willing to pay £150 per month to keep the **${lm://Field/1}** data private?

- I would pay £150 to keep the data private (1)
- I would not pay £150 to keep the data private (2)

Skip To: End of Block If ${lm://Field/1} Would you be willing to pay £150 per month to keep the ${lm://Field/1} data priva... = I would pay £150 to keep the data private

Skip To: End of Block If ${lm://Field/1} Would you be willing to pay £150 per month to keep the ${lm://Field/1} data priva... = I would not pay £150 to keep the data private

CondA_MedRec_100
${lm://Field/1}

Would you be willing to pay £100 per month to keep the **${lm://Field/1}** data private?

- I would pay £100 to keep the data private (1)
- I would not pay £100 to keep the data private (2)

Skip To: End of Block If ${lm://Field/1} Would you be willing to pay £100 per month to keep the ${lm://Field/1} data priva... = I would pay £100 to keep the data private

Skip To: End of Block If ${lm://Field/1} Would you be willing to pay £100 per month to keep the ${lm://Field/1} data priva... = I would not pay £100 to keep the data private

CondA_MedRec_50
${lm://Field/1}

Would you be willing to pay £50 per month to keep the **${lm://Field/1}** data private?

- I would pay £50 to keep the data private (1)
- I would not pay £50 to keep the data private (2)

Skip To: End of Block If ${lm://Field/1} Would you be willing to pay £50 per month to keep the ${lm://Field/1} data privat... = I would pay £50 to keep the data private

Skip To: End of Block If ${lm://Field/1} Would you be willing to pay £50 per month to keep the ${lm://Field/1} data privat... = I would not pay £50 to keep the data private

CondA_MedRec_30
${lm://Field/1}

Would you be willing to pay £30 per month to keep the **${lm://Field/1}** data private?

- I would pay £30 to keep the data private (1)
- I would not pay £30 to keep the data private (2)

Skip To: End of Block If ${lm://Field/1} Would you be willing to pay £30 per month to keep the ${lm://Field/1} data privat... = I would pay £30 to keep the data private

Skip To: End of Block If ${lm://Field/1} Would you be willing to pay £30 per month to keep the ${lm://Field/1} data privat... = I would not pay £30 to keep the data private

CondA_MedRec_20
${lm://Field/1}

Would you be willing to pay £20 per month to keep the **${lm://Field/1}** data private, or would you use the app for free and share the data?

- I would pay £20 to keep the data private (1)
- I would not pay £20 to keep the data private (2)

Skip To: End of Block If ${lm://Field/1} Would you be willing to pay £20 per month to keep the ${lm://Field/1} data privat... = I would pay £20 to keep the data private

Skip To: End of Block If ${lm://Field/1} Would you be willing to pay £20 per month to keep the ${lm://Field/1} data privat... = I would pay £20 to keep the data private

CondA_MedRec_10
${lm://Field/1}

Would you be willing to pay £10 per month to keep the **${lm://Field/1}** data private?

- I would pay £10 to keep the data private (1)
- I would not pay £10 to keep the data private (2)

Skip To: End of Block If ${lm://Field/1} Would you be willing to pay £10 per month to keep the ${lm://Field/1} data private? = I would pay £10 to keep the data private

Skip To: End of Block If ${lm://Field/1} Would you be willing to pay £10 per month to keep the ${lm://Field/1} data private? = I would not pay £10 to keep the data private

CondA_MedRec_1pound
${lm://Field/1}

Would you be willing to pay £1 per month to keep the **${lm://Field/1}** data private?

- I would pay £1 to keep the data private (1)
- I would not pay £1 to keep the data private (2)

Skip To: End of Block If ${lm://Field/1} Would you be willing to pay £1 per month to keep the ${lm://Field/1} data private... = I would pay £1 to keep the data private

Skip To: CondA_MedRec_1penny If ${lm://Field/1} Would you be willing to pay £1 per month to keep the ${lm://Field/1} data private... = I would not pay £1 to keep the data private

CondA_MedRec_1penny
${lm://Field/1}

Would you be willing to pay 1p per month to keep the **${lm://Field/1}** data private?

- I would pay 1p to keep the data private (1)
- I would not pay 1p to keep the data private (2)

Skip To: End of Block If ${lm://Field/1} Would you be willing to pay 1p per month to keep the ${lm://Field/1} data private?  = I would pay 1p to keep the data private

Skip To: End of Block If ${lm://Field/1} Would you be willing to pay 1p per month to keep the ${lm://Field/1} data private?  = I would not pay 1p to keep the data private

CondA_MedRec_MAX
${lm://Field/1}

You said you would be willing to pay £200 per month to keep the **${lm://Field/1}** data private. What is the maximum amount of money you would be willing to pay per month to keep this data private?

- Each month, I would pay up to £ (1) ________________________________________________

End of Block: CondA_MedRec

Start of Block: CondA_Loyalty Cards

CondA_Loyal_Intro **${lm://Field/1}**
When you first use the personal data app you are given two choices; either you can run the app in a “free” mode or or you can use it in “paid” mode. In “free” mode, you do not have to pay anything but the company providing the app will collect information about you when you use it. In “paid” mode, you pay money for the service and your data will remain private. If you choose this option, your data will not be used by the company providing the service for any purposes apart from maintaining the functionality of the app.

CondA_Loyal_Any Would you be willing to pay something, even a very small amount, to keep the **${lm://Field/1}** data private, or would you use the app for free and share the data with the app provider?

- I would pay something to keep the data private. (1)
- I would use the app for free and share the data. (2)

Skip To: CondA_Loyal_40 If Would you be willing to pay something, even a very small amount, to keep the ${lm://Field/1} data... = I would pay something to keep the data private.

Skip To: End of Block If Would you be willing to pay something, even a very small amount, to keep the ${lm://Field/1} data... = I would use the app for free and share the data.

CondA_Loyal_40
**${lm://Field/1}** 

Would you be willing to pay £40 per month to keep the **${lm://Field/1}** data private?

- I would pay £40 to keep the data private. (1)
- I would not pay £40 to keep the data private. (2)

Skip To: CondA_Loyal_125 If ${lm://Field/1}  Would you be willing to pay £40 per month to keep the ${lm://Field/1} data priva... = I would pay £40 to keep the data private.

Skip To: CondA_Loyal_15 If ${lm://Field/1}  Would you be willing to pay £40 per month to keep the ${lm://Field/1} data priva... = I would not pay £40 to keep the data private.

CondA_Loyal_125
**${lm://Field/1}** 

Would you be willing to pay £125 per month to keep the **${lm://Field/1}** data private?

- I would pay £125 to keep the data private. (1)
- I would not pay £125 to keep the data private. (2)

Skip To: CondA_Loyal_175 If ${lm://Field/1}  Would you be willing to pay £125 per month to keep the ${lm://Field/1} data priv... = I would pay £125 to keep the data private.

Skip To: CondA_Loyal_75 If ${lm://Field/1}  Would you be willing to pay £125 per month to keep the ${lm://Field/1} data priv... = I would not pay £125 to keep the data private.

CondA_Loyal_15
**${lm://Field/1}** 

Would you be willing to pay £15 per month to keep the **${lm://Field/1}** data private?

- I would pay £15 to keep the data private. (1)
- I would not pay £15 to keep the data private. (2)

Skip To: CondA_Loyal_25 If ${lm://Field/1}  Would you be willing to pay £15 per month to keep the ${lm://Field/1} data priva... = I would pay £15 to keep the data private.

Skip To: CondA_Loyal_5 If ${lm://Field/1}  Would you be willing to pay £15 per month to keep the ${lm://Field/1} data priva... = I would not pay £15 to keep the data private.

CondA_Loyal_175
**${lm://Field/1}** 

Would you be willing to pay £175 per month to keep the **${lm://Field/1}** data private?

- I would pay £175 to keep the data private (1)
- I would not pay £175 to keep the data private (2)

Skip To: CondA_Loyal_200 If ${lm://Field/1}  Would you be willing to pay £175 per month to keep the ${lm://Field/1} data priv... = I would pay £175 to keep the data private

Skip To: CondA_Loyal_150 If ${lm://Field/1}  Would you be willing to pay £175 per month to keep the ${lm://Field/1} data priv... = I would not pay £175 to keep the data private

CondA_Loyal_75
**${lm://Field/1}** 

Would you be willing to pay £75 per month to keep the **${lm://Field/1}** data private?

- I would pay £75 to keep the data private (1)
- I would not pay £75 to keep the data private (2)

Skip To: CondA_Loyal_100 If ${lm://Field/1}  Would you be willing to pay £75 per month to keep the ${lm://Field/1} data priva... = I would pay £75 to keep the data private

Skip To: CondA_Loyal_50 If ${lm://Field/1}  Would you be willing to pay £75 per month to keep the ${lm://Field/1} data priva... = I would not pay £75 to keep the data private

CondA_Loyal_25
**${lm://Field/1}** 

Would you be willing to pay £25 per month to keep the **${lm://Field/1}** data private?

- I would pay £25 to keep the data private (1)
- I would not pay £25 to keep the data private (2)

Skip To: CondA_Loyal_30 If ${lm://Field/1}  Would you be willing to pay £25 per month to keep the ${lm://Field/1} data priva... = I would pay £25 to keep the data private

Skip To: CondA_Loyal_20 If ${lm://Field/1}  Would you be willing to pay £25 per month to keep the ${lm://Field/1} data priva... = I would not pay £25 to keep the data private

CondA_Loyal_5
**${lm://Field/1}** 

Would you be willing to pay £5 per month to keep the **${lm://Field/1}** data private?

- I would pay £5 to keep the data private (1)
- I would not pay £5 to keep the data private (2)

Skip To: CondA_Loyal_10 If ${lm://Field/1}  Would you be willing to pay £5 per month to keep the ${lm://Field/1} data privat... = I would pay £5 to keep the data private

Skip To: CondA_Loyal_1pound If ${lm://Field/1}  Would you be willing to pay £5 per month to keep the ${lm://Field/1} data privat... = I would not pay £5 to keep the data private

CondA_Loyal_200
**${lm://Field/1}** 

Would you be willing to pay £200 per month to keep the **${lm://Field/1}** data private?

- I would pay £200 to keep the data private (1)
- I would not pay £200 to keep the data private (2)

Skip To: CondA_Loyal_Max If ${lm://Field/1}  Would you be willing to pay £200 per month to keep the ${lm://Field/1} data priv... = I would pay £200 to keep the data private

Skip To: End of Block If ${lm://Field/1}  Would you be willing to pay £200 per month to keep the ${lm://Field/1} data priv... = I would not pay £200 to keep the data private

CondA_Loyal_150
**${lm://Field/1}** 

Would you be willing to pay £150 per month to keep the **${lm://Field/1}** data private?

- I would pay £150 to keep the data private (1)
- I would not pay £150 to keep the data private (2)

Skip To: End of Block If ${lm://Field/1}  Would you be willing to pay £150 per month to keep the ${lm://Field/1} data priv... = I would pay £150 to keep the data private

Skip To: End of Block If ${lm://Field/1}  Would you be willing to pay £150 per month to keep the ${lm://Field/1} data priv... = I would not pay £150 to keep the data private

CondA_Loyal_100
**${lm://Field/1}** 

Would you be willing to pay £100 per month to keep the **${lm://Field/1}** data private?

- I would pay £100 to keep the data private (1)
- I would not pay £100 to keep the data private (2)

Skip To: End of Block If ${lm://Field/1}  Would you be willing to pay £100 per month to keep the ${lm://Field/1} data priv... = I would pay £100 to keep the data private

Skip To: End of Block If ${lm://Field/1}  Would you be willing to pay £100 per month to keep the ${lm://Field/1} data priv... = I would not pay £100 to keep the data private

CondA_Loyal_50
**${lm://Field/1}** 

Would you be willing to pay £50 per month to keep the **${lm://Field/1}** data private?

- I would pay £50 to keep the data private (1)
- I would not pay £50 to keep the data private (2)

Skip To: End of Block If ${lm://Field/1}  Would you be willing to pay £50 per month to keep the ${lm://Field/1} data priva... = I would pay £50 to keep the data private

Skip To: End of Block If ${lm://Field/1}  Would you be willing to pay £50 per month to keep the ${lm://Field/1} data priva... = I would not pay £50 to keep the data private

CondA_Loyal_30
**${lm://Field/1}** 

Would you be willing to pay £30 per month to keep the **${lm://Field/1}** data private?

- I would pay £30 to keep the data private (1)
- I would not pay £30 to keep the data private (2)

Skip To: End of Block If ${lm://Field/1}  Would you be willing to pay £30 per month to keep the ${lm://Field/1} data priva... = I would pay £30 to keep the data private

Skip To: End of Block If ${lm://Field/1}  Would you be willing to pay £30 per month to keep the ${lm://Field/1} data priva... = I would not pay £30 to keep the data private

CondA_Loyal_20
**${lm://Field/1}** 

Would you be willing to pay £20 per month to keep the **${lm://Field/1}** data private, or would you use the app for free and share the data?

- I would pay £20 to keep the data private (1)
- I would not pay £20 to keep the data private (2)

Skip To: End of Block If ${lm://Field/1}  Would you be willing to pay £20 per month to keep the ${lm://Field/1} data priva... = I would pay £20 to keep the data private

Skip To: End of Block If ${lm://Field/1}  Would you be willing to pay £20 per month to keep the ${lm://Field/1} data priva... = I would pay £20 to keep the data private

CondA_Loyal_10
**${lm://Field/1}** 

Would you be willing to pay £10 per month to keep the **${lm://Field/1}** data private?

- I would pay £10 to keep the data private (1)
- I would not pay £10 to keep the data private (2)

Skip To: End of Block If ${lm://Field/1}  Would you be willing to pay £10 per month to keep the ${lm://Field/1} data priva... = I would pay £10 to keep the data private

Skip To: End of Block If ${lm://Field/1}  Would you be willing to pay £10 per month to keep the ${lm://Field/1} data priva... = I would not pay £10 to keep the data private

CondA_Loyal_1pound
**${lm://Field/1}** 

Would you be willing to pay £1 per month to keep the **${lm://Field/1}** data private?

- I would pay £1 to keep the data private (1)
- I would not pay £1 to keep the data private (2)

Skip To: End of Block If ${lm://Field/1}  Would you be willing to pay £1 per month to keep the ${lm://Field/1} data privat... = I would pay £1 to keep the data private

Skip To: CondA_Loyal_1penny If ${lm://Field/1}  Would you be willing to pay £1 per month to keep the ${lm://Field/1} data privat... = I would not pay £1 to keep the data private

CondA_Loyal_1penny
**${lm://Field/1}** 

Would you be willing to pay 1p per month to keep the **${lm://Field/1}** data private?

- I would pay 1p to keep the data private (1)
- I would not pay 1p to keep the data private (2)

Skip To: End of Block If ${lm://Field/1}  Would you be willing to pay 1p per month to keep the ${lm://Field/1} data privat... = I would pay 1p to keep the data private

Skip To: End of Block If ${lm://Field/1}  Would you be willing to pay 1p per month to keep the ${lm://Field/1} data privat... = I would not pay 1p to keep the data private

CondA_Loyal_Max
**${lm://Field/1}** 

You said you would be willing to pay £200 per month to keep the **${lm://Field/1}** data private. What is the maximum amount of money you would be willing to pay per month to keep this data private?

- Each month, I would pay up to £ (1) ________________________________________________

End of Block: CondA_Loyalty Cards

Start of Block: CondA_Physical

CondA_Phys_Intro **${lm://Field/1}**
When you first use the personal data app you are given two choices; either you can run the app in a “free” mode or or you can use it in “paid” mode. In “free” mode, you do not have to pay anything but the company providing the app will collect information about you when you use it. In “paid” mode, you pay money for the service and your data will remain private. If you choose this option, your data will not be used by the company providing the service for any purposes apart from maintaining the functionality of the app.

CondA_Phys_Any Would you be willing to pay something, even a very small amount, to keep the **${lm://Field/1}** data private, or would you use the app for free and share the data with the app provider?

- I would pay something to keep the data private. (1)
- I would use the app for free and share the data. (2)

Skip To: CondA_Phys_40 If Would you be willing to pay something, even a very small amount, to keep the ${lm://Field/1} data... = I would pay something to keep the data private.

Skip To: End of Block If Would you be willing to pay something, even a very small amount, to keep the ${lm://Field/1} data... = I would use the app for free and share the data.

CondA_Phys_40
**${lm://Field/1}**

Would you be willing to pay £40 per month to keep the **${lm://Field/1}** data private?

- I would pay £40 to keep the data private. (1)
- I would not pay £40 to keep the data private. (2)

Skip To: CondA_Phys_125 If ${lm://Field/1} Would you be willing to pay £40 per month to keep the ${lm://Field/1} data privat... = I would pay £40 to keep the data private.

Skip To: CondA_Phys_15 If ${lm://Field/1} Would you be willing to pay £40 per month to keep the ${lm://Field/1} data privat... = I would not pay £40 to keep the data private.

CondA_Phys_125
**${lm://Field/1}**

Would you be willing to pay £125 per month to keep the **${lm://Field/1}** data private?

- I would pay £125 to keep the data private. (1)
- I would not pay £125 to keep the data private. (2)

Skip To: CondA_Phys_175 If ${lm://Field/1} Would you be willing to pay £125 per month to keep the ${lm://Field/1} data priva... = I would pay £125 to keep the data private.

Skip To: CondA_Phys_75 If ${lm://Field/1} Would you be willing to pay £125 per month to keep the ${lm://Field/1} data priva... = I would not pay £125 to keep the data private.

CondA_Phys_15
**${lm://Field/1}**

Would you be willing to pay £15 per month to keep the **${lm://Field/1}** data private?

- I would pay £15 to keep the data private. (1)
- I would not pay £15 to keep the data private. (2)

Skip To: CondA_Phys_25 If ${lm://Field/1} Would you be willing to pay £15 per month to keep the ${lm://Field/1} data privat... = I would pay £15 to keep the data private.

Skip To: CondA_Phys_5 If ${lm://Field/1} Would you be willing to pay £15 per month to keep the ${lm://Field/1} data privat... = I would not pay £15 to keep the data private.

CondA_Phys_175
**${lm://Field/1}**

Would you be willing to pay £175 per month to keep the **${lm://Field/1}** data private?

- I would pay £175 to keep the data private (1)
- I would not pay £175 to keep the data private (2)

Skip To: CondA_Phys_200 If ${lm://Field/1} Would you be willing to pay £175 per month to keep the ${lm://Field/1} data priva... = I would pay £175 to keep the data private

Skip To: CondA_Phys_150 If ${lm://Field/1} Would you be willing to pay £175 per month to keep the ${lm://Field/1} data priva... = I would not pay £175 to keep the data private

CondA_Phys_75
**${lm://Field/1}**

Would you be willing to pay £75 per month to keep the **${lm://Field/1}** data private?

- I would pay £75 to keep the data private (1)
- I would not pay £75 to keep the data private (2)

Skip To: CondA_Phys_100 If ${lm://Field/1} Would you be willing to pay £75 per month to keep the ${lm://Field/1} data privat... = I would pay £75 to keep the data private

Skip To: CondA_Phys_50 If ${lm://Field/1} Would you be willing to pay £75 per month to keep the ${lm://Field/1} data privat... = I would not pay £75 to keep the data private

CondA_Phys_25
**${lm://Field/1}**

Would you be willing to pay £25 per month to keep the **${lm://Field/1}** data private?

- I would pay £25 to keep the data private (1)
- I would not pay £25 to keep the data private (2)

Skip To: CondA_Phys_30 If ${lm://Field/1} Would you be willing to pay £25 per month to keep the ${lm://Field/1} data privat... = I would pay £25 to keep the data private

Skip To: CondA_Phys_20 If ${lm://Field/1} Would you be willing to pay £25 per month to keep the ${lm://Field/1} data privat... = I would not pay £25 to keep the data private

CondA_Phys_5
**${lm://Field/1}**

Would you be willing to pay £5 per month to keep the **${lm://Field/1}** data private?

- I would pay £5 to keep the data private (1)
- I would not pay £5 to keep the data private (2)

Skip To: CondA_Phys_10 If ${lm://Field/1} Would you be willing to pay £5 per month to keep the ${lm://Field/1} data private... = I would pay £5 to keep the data private

Skip To: CondA_Phys_1pound If ${lm://Field/1} Would you be willing to pay £5 per month to keep the ${lm://Field/1} data private... = I would not pay £5 to keep the data private

CondA_Phys_200
**${lm://Field/1}**

Would you be willing to pay £200 per month to keep the **${lm://Field/1}** data private?

- I would pay £200 to keep the data private (1)
- I would not pay £200 to keep the data private (2)

Skip To: CondA_Phys_Max If ${lm://Field/1} Would you be willing to pay £200 per month to keep the ${lm://Field/1} data priva... = I would pay £200 to keep the data private

Skip To: End of Block If ${lm://Field/1} Would you be willing to pay £200 per month to keep the ${lm://Field/1} data priva... = I would not pay £200 to keep the data private

CondA_Phys_150
**${lm://Field/1}**

Would you be willing to pay £150 per month to keep the **${lm://Field/1}** data private?

- I would pay £150 to keep the data private (1)
- I would not pay £150 to keep the data private (2)

Skip To: End of Block If ${lm://Field/1} Would you be willing to pay £150 per month to keep the ${lm://Field/1} data priva... = I would pay £150 to keep the data private

Skip To: End of Block If ${lm://Field/1} Would you be willing to pay £150 per month to keep the ${lm://Field/1} data priva... = I would not pay £150 to keep the data private

CondA_Phys_100
**${lm://Field/1}**

Would you be willing to pay £100 per month to keep the **${lm://Field/1}** data private?

- I would pay £100 to keep the data private (1)
- I would not pay £100 to keep the data private (2)

Skip To: End of Block If ${lm://Field/1} Would you be willing to pay £100 per month to keep the ${lm://Field/1} data priva... = I would pay £100 to keep the data private

Skip To: End of Block If ${lm://Field/1} Would you be willing to pay £100 per month to keep the ${lm://Field/1} data priva... = I would not pay £100 to keep the data private

CondA_Phys_50
**${lm://Field/1}**

Would you be willing to pay £50 per month to keep the **${lm://Field/1}** data private?

- I would pay £50 to keep the data private (1)
- I would not pay £50 to keep the data private (2)

Skip To: End of Block If ${lm://Field/1} Would you be willing to pay £50 per month to keep the ${lm://Field/1} data privat... = I would pay £50 to keep the data private

Skip To: End of Block If ${lm://Field/1} Would you be willing to pay £50 per month to keep the ${lm://Field/1} data privat... = I would not pay £50 to keep the data private

CondA_Phys_30
**${lm://Field/1}**

Would you be willing to pay £30 per month to keep the **${lm://Field/1}** data private?

- I would pay £30 to keep the data private (1)
- I would not pay £30 to keep the data private (2)

Skip To: End of Block If ${lm://Field/1} Would you be willing to pay £30 per month to keep the ${lm://Field/1} data privat... = I would pay £30 to keep the data private

Skip To: End of Block If ${lm://Field/1} Would you be willing to pay £30 per month to keep the ${lm://Field/1} data privat... = I would not pay £30 to keep the data private

CondA_Phys_20
**${lm://Field/1}**

Would you be willing to pay £20 per month to keep the **${lm://Field/1}** data private, or would you use the app for free and share the data?

- I would pay £20 to keep the data private (1)
- I would not pay £20 to keep the data private (2)

Skip To: End of Block If ${lm://Field/1} Would you be willing to pay £20 per month to keep the ${lm://Field/1} data privat... = I would pay £20 to keep the data private

Skip To: End of Block If ${lm://Field/1} Would you be willing to pay £20 per month to keep the ${lm://Field/1} data privat... = I would pay £20 to keep the data private

CondA_Phys_10
**${lm://Field/1}**

Would you be willing to pay £10 per month to keep the **${lm://Field/1}** data private?

- I would pay £10 to keep the data private (1)
- I would not pay £10 to keep the data private (2)

Skip To: End of Block If ${lm://Field/1} Would you be willing to pay £10 per month to keep the ${lm://Field/1} data private? = I would pay £10 to keep the data private

Skip To: End of Block If ${lm://Field/1} Would you be willing to pay £10 per month to keep the ${lm://Field/1} data private? = I would not pay £10 to keep the data private

CondA_Phys_1pound
**${lm://Field/1}**

Would you be willing to pay £1 per month to keep the **${lm://Field/1}** data private?

- I would pay £1 to keep the data private (1)
- I would not pay £1 to keep the data private (2)

Skip To: End of Block If ${lm://Field/1} Would you be willing to pay £1 per month to keep the ${lm://Field/1} data private... = I would pay £1 to keep the data private

Skip To: CondA_Phys_1penny If ${lm://Field/1} Would you be willing to pay £1 per month to keep the ${lm://Field/1} data private... = I would not pay £1 to keep the data private

CondA_Phys_1penny
**${lm://Field/1}**

Would you be willing to pay 1p per month to keep the **${lm://Field/1}** data private?

- I would pay 1p to keep the data private (1)
- I would not pay 1p to keep the data private (2)

Skip To: End of Block If ${lm://Field/1} Would you be willing to pay 1p per month to keep the ${lm://Field/1} data private?  = I would pay 1p to keep the data private

Skip To: End of Block If ${lm://Field/1} Would you be willing to pay 1p per month to keep the ${lm://Field/1} data private?  = I would not pay 1p to keep the data private

CondA_Phys_Max
**${lm://Field/1}**

You said you would be willing to pay £200 per month to keep the **${lm://Field/1}** data private. What is the maximum amount of money you would be willing to pay per month to keep this data private?

- Each month, I would pay up to £ (1) ________________________________________________

End of Block: CondA_Physical

Start of Block: CondA_Social

CondA_Social_Intro **${lm://Field/1}**
When you first use the personal data app you are given two choices; either you can run the app in a “free” mode or or you can use it in “paid” mode. In “free” mode, you do not have to pay anything but the company providing the app will collect information about you when you use it. In “paid” mode, you pay money for the service and your data will remain private. If you choose this option, your data will not be used by the company providing the service for any purposes apart from maintaining the functionality of the app.

CondA_Social_Any Would you be willing to pay something, even a very small amount, to keep the **${lm://Field/1}** data private, or would you use the app for free and share the data with the app provider?

- I would pay something to keep the data private. (1)
- I would use the app for free and share the data. (2)

Skip To: CondA_Social_40 If Would you be willing to pay something, even a very small amount, to keep the ${lm://Field/1} data... = I would pay something to keep the data private.

Skip To: End of Block If Would you be willing to pay something, even a very small amount, to keep the ${lm://Field/1} data... = I would use the app for free and share the data.

CondA_Social_40
**${lm://Field/1}**

Would you be willing to pay £40 per month to keep the **${lm://Field/1}** data private?

- I would pay £40 to keep the data private. (1)
- I would not pay £40 to keep the data private. (2)

Skip To: CondA_Social_125 If ${lm://Field/1} Would you be willing to pay £40 per month to keep the ${lm://Field/1} data privat... = I would pay £40 to keep the data private.

Skip To: CondA_Social_15 If ${lm://Field/1} Would you be willing to pay £40 per month to keep the ${lm://Field/1} data privat... = I would not pay £40 to keep the data private.

CondA_Social_125
**${lm://Field/1}**

Would you be willing to pay £125 per month to keep the **${lm://Field/1}** data private?

- I would pay £125 to keep the data private. (1)
- I would not pay £125 to keep the data private. (2)

Skip To: CondA_Social_175 If ${lm://Field/1} Would you be willing to pay £125 per month to keep the ${lm://Field/1} data priva... = I would pay £125 to keep the data private.

Skip To: CondA_Social_75 If ${lm://Field/1} Would you be willing to pay £125 per month to keep the ${lm://Field/1} data priva... = I would not pay £125 to keep the data private.

CondA_Social_15
**${lm://Field/1}**

Would you be willing to pay £15 per month to keep the **${lm://Field/1}** data private?

- I would pay £15 to keep the data private. (1)
- I would not pay £15 to keep the data private. (2)

Skip To: CondA_Social_25 If ${lm://Field/1} Would you be willing to pay £15 per month to keep the ${lm://Field/1} data privat... = I would pay £15 to keep the data private.

Skip To: CondA_Social_5 If ${lm://Field/1} Would you be willing to pay £15 per month to keep the ${lm://Field/1} data privat... = I would not pay £15 to keep the data private.

CondA_Social_175
**${lm://Field/1}**

Would you be willing to pay £175 per month to keep the **${lm://Field/1}** data private?

- I would pay £175 to keep the data private (1)
- I would not pay £175 to keep the data private (2)

Skip To: CondA_Social_200 If ${lm://Field/1} Would you be willing to pay £175 per month to keep the ${lm://Field/1} data priva... = I would pay £175 to keep the data private

Skip To: CondA_Social_150 If ${lm://Field/1} Would you be willing to pay £175 per month to keep the ${lm://Field/1} data priva... = I would not pay £175 to keep the data private

CondA_Social_75
**${lm://Field/1}**

Would you be willing to pay £75 per month to keep the **${lm://Field/1}** data private?

- I would pay £75 to keep the data private (1)
- I would not pay £75 to keep the data private (2)

Skip To: CondA_Social_100 If ${lm://Field/1} Would you be willing to pay £75 per month to keep the ${lm://Field/1} data privat... = I would pay £75 to keep the data private

Skip To: CondA_Social_50 If ${lm://Field/1} Would you be willing to pay £75 per month to keep the ${lm://Field/1} data privat... = I would not pay £75 to keep the data private

CondA_Social_25
**${lm://Field/1}**

Would you be willing to pay £25 per month to keep the **${lm://Field/1}** data private?

- I would pay £25 to keep the data private (1)
- I would not pay £25 to keep the data private (2)

Skip To: CondA_Social_30 If ${lm://Field/1} Would you be willing to pay £25 per month to keep the ${lm://Field/1} data privat... = I would pay £25 to keep the data private

Skip To: CondA_Social_20 If ${lm://Field/1} Would you be willing to pay £25 per month to keep the ${lm://Field/1} data privat... = I would not pay £25 to keep the data private

CondA_Social_5
**${lm://Field/1}**

Would you be willing to pay £5 per month to keep the **${lm://Field/1}** data private?

- I would pay £5 to keep the data private (1)
- I would not pay £5 to keep the data private (2)

Skip To: CondA_Social_10 If ${lm://Field/1} Would you be willing to pay £5 per month to keep the ${lm://Field/1} data private... = I would pay £5 to keep the data private

Skip To: CondA_Social_1pound If ${lm://Field/1} Would you be willing to pay £5 per month to keep the ${lm://Field/1} data private... = I would not pay £5 to keep the data private

CondA_Social_200
**${lm://Field/1}**

Would you be willing to pay £200 per month to keep the **${lm://Field/1}** data private?

- I would pay £200 to keep the data private (1)
- I would not pay £200 to keep the data private (2)

Skip To: CondA_Social_Max If ${lm://Field/1} Would you be willing to pay £200 per month to keep the ${lm://Field/1} data priva... = I would pay £200 to keep the data private

Skip To: End of Block If ${lm://Field/1} Would you be willing to pay £200 per month to keep the ${lm://Field/1} data priva... = I would not pay £200 to keep the data private

CondA_Social_150
**${lm://Field/1}**

Would you be willing to pay £150 per month to keep the **${lm://Field/1}** data private?

- I would pay £150 to keep the data private (1)
- I would not pay £150 to keep the data private (2)

Skip To: End of Block If ${lm://Field/1} Would you be willing to pay £150 per month to keep the ${lm://Field/1} data priva... = I would pay £150 to keep the data private

Skip To: End of Block If ${lm://Field/1} Would you be willing to pay £150 per month to keep the ${lm://Field/1} data priva... = I would not pay £150 to keep the data private

CondA_Social_100
**${lm://Field/1}**

Would you be willing to pay £100 per month to keep the **${lm://Field/1}** data private?

- I would pay £100 to keep the data private (1)
- I would not pay £100 to keep the data private (2)

Skip To: End of Block If ${lm://Field/1} Would you be willing to pay £100 per month to keep the ${lm://Field/1} data priva... = I would pay £100 to keep the data private

Skip To: End of Block If ${lm://Field/1} Would you be willing to pay £100 per month to keep the ${lm://Field/1} data priva... = I would not pay £100 to keep the data private

CondA_Social_50
**${lm://Field/1}**

Would you be willing to pay £50 per month to keep the **${lm://Field/1}** data private?

- I would pay £50 to keep the data private (1)
- I would not pay £50 to keep the data private (2)

Skip To: End of Block If ${lm://Field/1} Would you be willing to pay £50 per month to keep the ${lm://Field/1} data privat... = I would pay £50 to keep the data private

Skip To: End of Block If ${lm://Field/1} Would you be willing to pay £50 per month to keep the ${lm://Field/1} data privat... = I would not pay £50 to keep the data private

CondA_Social_30
**${lm://Field/1}**

Would you be willing to pay £30 per month to keep the **${lm://Field/1}** data private?

- I would pay £30 to keep the data private (1)
- I would not pay £30 to keep the data private (2)

Skip To: End of Block If ${lm://Field/1} Would you be willing to pay £30 per month to keep the ${lm://Field/1} data privat... = I would pay £30 to keep the data private

Skip To: End of Block If ${lm://Field/1} Would you be willing to pay £30 per month to keep the ${lm://Field/1} data privat... = I would not pay £30 to keep the data private

CondA_Social_20
**${lm://Field/1}**

Would you be willing to pay £20 per month to keep the **${lm://Field/1}** data private, or would you use the app for free and share the data?

- I would pay £20 to keep the data private (1)
- I would not pay £20 to keep the data private (2)

Skip To: End of Block If ${lm://Field/1} Would you be willing to pay £20 per month to keep the ${lm://Field/1} data privat... = I would pay £20 to keep the data private

Skip To: End of Block If ${lm://Field/1} Would you be willing to pay £20 per month to keep the ${lm://Field/1} data privat... = I would pay £20 to keep the data private

CondA_Social_10
**${lm://Field/1}**

Would you be willing to pay £10 per month to keep the **${lm://Field/1}** data private?

- I would pay £10 to keep the data private (1)
- I would not pay £10 to keep the data private (2)

Skip To: End of Block If ${lm://Field/1} Would you be willing to pay £10 per month to keep the ${lm://Field/1} data private? = I would pay £10 to keep the data private

Skip To: End of Block If ${lm://Field/1} Would you be willing to pay £10 per month to keep the ${lm://Field/1} data private? = I would not pay £10 to keep the data private

CondA_Social_1pound
**${lm://Field/1}**

Would you be willing to pay £1 per month to keep the **${lm://Field/1}** data private?

- I would pay £1 to keep the data private (1)
- I would not pay £1 to keep the data private (2)

Skip To: End of Block If ${lm://Field/1} Would you be willing to pay £1 per month to keep the ${lm://Field/1} data private... = I would pay £1 to keep the data private

Skip To: CondA_Social_1penny If ${lm://Field/1} Would you be willing to pay £1 per month to keep the ${lm://Field/1} data private... = I would not pay £1 to keep the data private

CondA_Social_1penny
**${lm://Field/1}**

Would you be willing to pay 1p per month to keep the **${lm://Field/1}** data private?

- I would pay 1p to keep the data private (1)
- I would not pay 1p to keep the data private (2)

Skip To: End of Block If ${lm://Field/1} Would you be willing to pay 1p per month to keep the ${lm://Field/1} data private?  = I would pay 1p to keep the data private

Skip To: End of Block If ${lm://Field/1} Would you be willing to pay 1p per month to keep the ${lm://Field/1} data private?  = I would not pay 1p to keep the data private

CondA_Social_Max
**${lm://Field/1}**

You said you would be willing to pay £200 per month to keep the **${lm://Field/1}** data private. What is the maximum amount of money you would be willing to pay per month to keep this data private?

- Each month, I would pay up to £ (1) ________________________________________________

End of Block: CondA_Social

Start of Block: CondA_Electricity

CondA_Elec_Intro **${lm://Field/1}**
When you first use the personal data app you are given two choices; either you can run the app in a “free” mode or or you can use it in “paid” mode. In “free” mode, you do not have to pay anything but the company providing the app will collect information about you when you use it. In “paid” mode, you pay money for the service and your data will remain private. If you choose this option, your data will not be used by the company providing the service for any purposes apart from maintaining the functionality of the app.

CondA_Elec_Any Would you be willing to pay something, even a very small amount, to keep the **${lm://Field/1}** data private, or would you use the app for free and share the data with the app provider?

- I would pay something to keep the data private. (1)
- I would use the app for free and share the data. (2)

Skip To: CondA_Elec_40 If Would you be willing to pay something, even a very small amount, to keep the ${lm://Field/1} data... = I would pay something to keep the data private.

Skip To: End of Block If Would you be willing to pay something, even a very small amount, to keep the ${lm://Field/1} data... = I would use the app for free and share the data.

CondA_Elec_40
**${lm://Field/1}**

Would you be willing to pay £40 per month to keep the **${lm://Field/1}** data private?

- I would pay £40 to keep the data private. (1)
- I would not pay £40 to keep the data private. (2)

Skip To: CondA_Elec_125 If ${lm://Field/1} Would you be willing to pay £40 per month to keep the ${lm://Field/1} data privat... = I would pay £40 to keep the data private.

Skip To: CondA_Elec_15 If ${lm://Field/1} Would you be willing to pay £40 per month to keep the ${lm://Field/1} data privat... = I would not pay £40 to keep the data private.

CondA_Elec_125
**${lm://Field/1}**

Would you be willing to pay £125 per month to keep the **${lm://Field/1}** data private?

- I would pay £125 to keep the data private. (1)
- I would not pay £125 to keep the data private. (2)

Skip To: CondA_Elec_175 If ${lm://Field/1} Would you be willing to pay £125 per month to keep the ${lm://Field/1} data priva... = I would pay £125 to keep the data private.

Skip To: CondA_Elec_75 If ${lm://Field/1} Would you be willing to pay £125 per month to keep the ${lm://Field/1} data priva... = I would not pay £125 to keep the data private.

CondA_Elec_15
**${lm://Field/1}**

Would you be willing to pay £15 per month to keep the **${lm://Field/1}** data private?

- I would pay £15 to keep the data private. (1)
- I would not pay £15 to keep the data private. (2)

Skip To: CondA_Elec_25 If ${lm://Field/1} Would you be willing to pay £15 per month to keep the ${lm://Field/1} data privat... = I would pay £15 to keep the data private.

Skip To: CondA_Elec_5 If ${lm://Field/1} Would you be willing to pay £15 per month to keep the ${lm://Field/1} data privat... = I would not pay £15 to keep the data private.

CondA_Elec_175
**${lm://Field/1}**

Would you be willing to pay £175 per month to keep the **${lm://Field/1}** data private?

- I would pay £175 to keep the data private (1)
- I would not pay £175 to keep the data private (2)

Skip To: CondA_Elec_200 If ${lm://Field/1} Would you be willing to pay £175 per month to keep the ${lm://Field/1} data priva... = I would pay £175 to keep the data private

Skip To: CondA_Elec_150 If ${lm://Field/1} Would you be willing to pay £175 per month to keep the ${lm://Field/1} data priva... = I would not pay £175 to keep the data private

CondA_Elec_75
**${lm://Field/1}**

Would you be willing to pay £75 per month to keep the **${lm://Field/1}** data private?

- I would pay £75 to keep the data private (1)
- I would not pay £75 to keep the data private (2)

Skip To: CondA_Elec_100 If ${lm://Field/1} Would you be willing to pay £75 per month to keep the ${lm://Field/1} data privat... = I would pay £75 to keep the data private

Skip To: CondA_Elec_50 If ${lm://Field/1} Would you be willing to pay £75 per month to keep the ${lm://Field/1} data privat... = I would not pay £75 to keep the data private

CondA_Elec_25
**${lm://Field/1}**

Would you be willing to pay £25 per month to keep the **${lm://Field/1}** data private?

- I would pay £25 to keep the data private (1)
- I would not pay £25 to keep the data private (2)

Skip To: CondA_Elec_30 If ${lm://Field/1} Would you be willing to pay £25 per month to keep the ${lm://Field/1} data privat... = I would pay £25 to keep the data private

Skip To: CondA_Elec_20 If ${lm://Field/1} Would you be willing to pay £25 per month to keep the ${lm://Field/1} data privat... = I would not pay £25 to keep the data private

CondA_Elec_5
**${lm://Field/1}**

Would you be willing to pay £5 per month to keep the **${lm://Field/1}** data private?

- I would pay £5 to keep the data private (1)
- I would not pay £5 to keep the data private (2)

Skip To: CondA_Elec_10 If ${lm://Field/1} Would you be willing to pay £5 per month to keep the ${lm://Field/1} data private... = I would pay £5 to keep the data private

Skip To: CondA_Elec_1pound If ${lm://Field/1} Would you be willing to pay £5 per month to keep the ${lm://Field/1} data private... = I would not pay £5 to keep the data private

CondA_Elec_200
**${lm://Field/1}**

Would you be willing to pay £200 per month to keep the **${lm://Field/1}** data private?

- I would pay £200 to keep the data private (1)
- I would not pay £200 to keep the data private (2)

Skip To: CondA_Elec_Max If ${lm://Field/1} Would you be willing to pay £200 per month to keep the ${lm://Field/1} data priva... = I would pay £200 to keep the data private

Skip To: End of Block If ${lm://Field/1} Would you be willing to pay £200 per month to keep the ${lm://Field/1} data priva... = I would not pay £200 to keep the data private

CondA_Elec_150
**${lm://Field/1}**

Would you be willing to pay £150 per month to keep the **${lm://Field/1}** data private?

- I would pay £150 to keep the data private (1)
- I would not pay £150 to keep the data private (2)

Skip To: End of Block If ${lm://Field/1} Would you be willing to pay £150 per month to keep the ${lm://Field/1} data priva... = I would pay £150 to keep the data private

Skip To: End of Block If ${lm://Field/1} Would you be willing to pay £150 per month to keep the ${lm://Field/1} data priva... = I would not pay £150 to keep the data private

CondA_Elec_100
**${lm://Field/1}**

Would you be willing to pay £100 per month to keep the **${lm://Field/1}** data private?

- I would pay £100 to keep the data private (1)
- I would not pay £100 to keep the data private (2)

Skip To: End of Block If ${lm://Field/1} Would you be willing to pay £100 per month to keep the ${lm://Field/1} data priva... = I would pay £100 to keep the data private

Skip To: End of Block If ${lm://Field/1} Would you be willing to pay £100 per month to keep the ${lm://Field/1} data priva... = I would not pay £100 to keep the data private

CondA_Elec_50
**${lm://Field/1}**

Would you be willing to pay £50 per month to keep the **${lm://Field/1}** data private?

- I would pay £50 to keep the data private (1)
- I would not pay £50 to keep the data private (2)

Skip To: End of Block If ${lm://Field/1} Would you be willing to pay £50 per month to keep the ${lm://Field/1} data privat... = I would pay £50 to keep the data private

Skip To: End of Block If ${lm://Field/1} Would you be willing to pay £50 per month to keep the ${lm://Field/1} data privat... = I would not pay £50 to keep the data private

CondA_Elec_30
**${lm://Field/1}**

Would you be willing to pay £30 per month to keep the **${lm://Field/1}** data private?

- I would pay £30 to keep the data private (1)
- I would not pay £30 to keep the data private (2)

Skip To: End of Block If ${lm://Field/1} Would you be willing to pay £30 per month to keep the ${lm://Field/1} data privat... = I would pay £30 to keep the data private

Skip To: End of Block If ${lm://Field/1} Would you be willing to pay £30 per month to keep the ${lm://Field/1} data privat... = I would not pay £30 to keep the data private

CondA_Elec_20
**${lm://Field/1}**

Would you be willing to pay £20 per month to keep the **${lm://Field/1}** data private, or would you use the app for free and share the data?

- I would pay £20 to keep the data private (1)
- I would not pay £20 to keep the data private (2)

Skip To: End of Block If ${lm://Field/1} Would you be willing to pay £20 per month to keep the ${lm://Field/1} data privat... = I would pay £20 to keep the data private

Skip To: End of Block If ${lm://Field/1} Would you be willing to pay £20 per month to keep the ${lm://Field/1} data privat... = I would pay £20 to keep the data private

CondA_Elec_10
**${lm://Field/1}**

Would you be willing to pay £10 per month to keep the **${lm://Field/1}** data private?

- I would pay £10 to keep the data private (1)
- I would not pay £10 to keep the data private (2)

Skip To: End of Block If ${lm://Field/1} Would you be willing to pay £10 per month to keep the ${lm://Field/1} data private? = I would pay £10 to keep the data private

Skip To: End of Block If ${lm://Field/1} Would you be willing to pay £10 per month to keep the ${lm://Field/1} data private? = I would not pay £10 to keep the data private

CondA_Elec_1pound
**${lm://Field/1}**

Would you be willing to pay £1 per month to keep the **${lm://Field/1}** data private?

- I would pay £1 to keep the data private (1)
- I would not pay £1 to keep the data private (2)

Skip To: End of Block If ${lm://Field/1} Would you be willing to pay £1 per month to keep the ${lm://Field/1} data private... = I would pay £1 to keep the data private

Skip To: CondA_Elec_1penny If ${lm://Field/1} Would you be willing to pay £1 per month to keep the ${lm://Field/1} data private... = I would not pay £1 to keep the data private

CondA_Elec_1penny
**${lm://Field/1}**

Would you be willing to pay 1p per month to keep the **${lm://Field/1}** data private?

- I would pay 1p to keep the data private (1)
- I would not pay 1p to keep the data private (2)

Skip To: End of Block If ${lm://Field/1} Would you be willing to pay 1p per month to keep the ${lm://Field/1} data private?  = I would pay 1p to keep the data private

Skip To: End of Block If ${lm://Field/1} Would you be willing to pay 1p per month to keep the ${lm://Field/1} data private?  = I would not pay 1p to keep the data private

CondA_Elec_Max
**${lm://Field/1}**

You said you would be willing to pay £200 per month to keep the **${lm://Field/1}** data private. What is the maximum amount of money you would be willing to pay per month to keep this data private?

- Each month, I would pay up to £ (1) ________________________________________________

End of Block: CondA_Electricity

Start of Block: CondA_Mobile GPS

CondA_MoGPS_Intro **${lm://Field/1}**
When you first use the personal data app you are given two choices; either you can run the app in a “free” mode or or you can use it in “paid” mode. In “free” mode, you do not have to pay anything but the company providing the app will collect information about you when you use it. In “paid” mode, you pay money for the service and your data will remain private. If you choose this option, your data will not be used by the company providing the service for any purposes apart from maintaining the functionality of the app.

CondA_MoGPS_Any Would you be willing to pay something, even a very small amount, to keep the **${lm://Field/1}** data private, or would you use the app for free and share the data with the app provider?

- I would pay something to keep the data private. (1)
- I would use the app for free and share the data. (2)

Skip To: CondA_MoGPS_40 If Would you be willing to pay something, even a very small amount, to keep the ${lm://Field/1} data... = I would pay something to keep the data private.

Skip To: End of Block If Would you be willing to pay something, even a very small amount, to keep the ${lm://Field/1} data... = I would use the app for free and share the data.

CondA_MoGPS_40
${lm://Field/1}

Would you be willing to pay £40 per month to keep the **${lm://Field/1}** data private?

- I would pay £40 to keep the data private. (1)
- I would not pay £40 to keep the data private. (2)

Skip To: CondA_MoGPS_125 If ${lm://Field/1} Would you be willing to pay £40 per month to keep the ${lm://Field/1} data privat... = I would pay £40 to keep the data private.

Skip To: CondA_MoGPS_15 If ${lm://Field/1} Would you be willing to pay £40 per month to keep the ${lm://Field/1} data privat... = I would not pay £40 to keep the data private.

CondA_MoGPS_125
${lm://Field/1}

Would you be willing to pay £125 per month to keep the **${lm://Field/1}** data private?

- I would pay £125 to keep the data private. (1)
- I would not pay £125 to keep the data private. (2)

Skip To: CondA_MoGPS_175 If ${lm://Field/1} Would you be willing to pay £125 per month to keep the ${lm://Field/1} data priva... = I would pay £125 to keep the data private.

Skip To: CondA_MoGPS_75 If ${lm://Field/1} Would you be willing to pay £125 per month to keep the ${lm://Field/1} data priva... = I would not pay £125 to keep the data private.

CondA_MoGPS_15
${lm://Field/1}

Would you be willing to pay £15 per month to keep the **${lm://Field/1}** data private?

- I would pay £15 to keep the data private. (1)
- I would not pay £15 to keep the data private. (2)

Skip To: CondA_MoGPS_25 If ${lm://Field/1} Would you be willing to pay £15 per month to keep the ${lm://Field/1} data privat... = I would pay £15 to keep the data private.

Skip To: CondA_MoGPS_5 If ${lm://Field/1} Would you be willing to pay £15 per month to keep the ${lm://Field/1} data privat... = I would not pay £15 to keep the data private.

CondA_MoGPS_175
${lm://Field/1}

Would you be willing to pay £175 per month to keep the **${lm://Field/1}** data private?

- I would pay £175 to keep the data private (1)
- I would not pay £175 to keep the data private (2)

Skip To: CondA_MoGPS_200 If ${lm://Field/1} Would you be willing to pay £175 per month to keep the ${lm://Field/1} data priva... = I would pay £175 to keep the data private

Skip To: CondA_MoGPS_150 If ${lm://Field/1} Would you be willing to pay £175 per month to keep the ${lm://Field/1} data priva... = I would not pay £175 to keep the data private

CondA_MoGPS_75
${lm://Field/1}

Would you be willing to pay £75 per month to keep the **${lm://Field/1}** data private?

- I would pay £75 to keep the data private (1)
- I would not pay £75 to keep the data private (2)

Skip To: CondA_MoGPS_100 If ${lm://Field/1} Would you be willing to pay £75 per month to keep the ${lm://Field/1} data privat... = I would pay £75 to keep the data private

Skip To: CondA_MoGPS_50 If ${lm://Field/1} Would you be willing to pay £75 per month to keep the ${lm://Field/1} data privat... = I would not pay £75 to keep the data private

CondA_MoGPS_25
${lm://Field/1}

Would you be willing to pay £25 per month to keep the **${lm://Field/1}** data private?

- I would pay £25 to keep the data private (1)
- I would not pay £25 to keep the data private (2)

Skip To: CondA_MoGPS_30 If ${lm://Field/1} Would you be willing to pay £25 per month to keep the ${lm://Field/1} data privat... = I would pay £25 to keep the data private

Skip To: CondA_MoGPS_20 If ${lm://Field/1} Would you be willing to pay £25 per month to keep the ${lm://Field/1} data privat... = I would not pay £25 to keep the data private

CondA_MoGPS_5
${lm://Field/1}

Would you be willing to pay £5 per month to keep the **${lm://Field/1}** data private?

- I would pay £5 to keep the data private (1)
- I would not pay £5 to keep the data private (2)

Skip To: CondA_MoGPS_10 If ${lm://Field/1} Would you be willing to pay £5 per month to keep the ${lm://Field/1} data private... = I would pay £5 to keep the data private

Skip To: CondA_MoGPS_1pound If ${lm://Field/1} Would you be willing to pay £5 per month to keep the ${lm://Field/1} data private... = I would not pay £5 to keep the data private

CondA_MoGPS_200
${lm://Field/1}

Would you be willing to pay £200 per month to keep the **${lm://Field/1}** data private?

- I would pay £200 to keep the data private (1)
- I would not pay £200 to keep the data private (2)

Skip To: CondA_MoGPS_Max If ${lm://Field/1} Would you be willing to pay £200 per month to keep the ${lm://Field/1} data priva... = I would pay £200 to keep the data private

Skip To: End of Block If ${lm://Field/1} Would you be willing to pay £200 per month to keep the ${lm://Field/1} data priva... = I would not pay £200 to keep the data private

CondA_MoGPS_150
${lm://Field/1}

Would you be willing to pay £150 per month to keep the **${lm://Field/1}** data private?

- I would pay £150 to keep the data private (1)
- I would not pay £150 to keep the data private (2)

Skip To: End of Block If ${lm://Field/1} Would you be willing to pay £150 per month to keep the ${lm://Field/1} data priva... = I would pay £150 to keep the data private

Skip To: End of Block If ${lm://Field/1} Would you be willing to pay £150 per month to keep the ${lm://Field/1} data priva... = I would not pay £150 to keep the data private

CondA_MoGPS_100
${lm://Field/1}

Would you be willing to pay £100 per month to keep the **${lm://Field/1}** data private?

- I would pay £100 to keep the data private (1)
- I would not pay £100 to keep the data private (2)

Skip To: End of Block If ${lm://Field/1} Would you be willing to pay £100 per month to keep the ${lm://Field/1} data priva... = I would pay £100 to keep the data private

Skip To: End of Block If ${lm://Field/1} Would you be willing to pay £100 per month to keep the ${lm://Field/1} data priva... = I would not pay £100 to keep the data private

CondA_MoGPS_50
${lm://Field/1}

Would you be willing to pay £50 per month to keep the **${lm://Field/1}** data private?

- I would pay £50 to keep the data private (1)
- I would not pay £50 to keep the data private (2)

Skip To: End of Block If ${lm://Field/1} Would you be willing to pay £50 per month to keep the ${lm://Field/1} data privat... = I would pay £50 to keep the data private

Skip To: End of Block If ${lm://Field/1} Would you be willing to pay £50 per month to keep the ${lm://Field/1} data privat... = I would not pay £50 to keep the data private

CondA_MoGPS_30
${lm://Field/1}

Would you be willing to pay £30 per month to keep the **${lm://Field/1}** data private?

- I would pay £30 to keep the data private (1)
- I would not pay £30 to keep the data private (2)

Skip To: End of Block If ${lm://Field/1} Would you be willing to pay £30 per month to keep the ${lm://Field/1} data privat... = I would pay £30 to keep the data private

Skip To: End of Block If ${lm://Field/1} Would you be willing to pay £30 per month to keep the ${lm://Field/1} data privat... = I would not pay £30 to keep the data private

CondA_MoGPS_20
${lm://Field/1}

Would you be willing to pay £20 per month to keep the **${lm://Field/1}** data private, or would you use the app for free and share the data?

- I would pay £20 to keep the data private (1)
- I would not pay £20 to keep the data private (2)

Skip To: End of Block If ${lm://Field/1} Would you be willing to pay £20 per month to keep the ${lm://Field/1} data privat... = I would pay £20 to keep the data private

Skip To: End of Block If ${lm://Field/1} Would you be willing to pay £20 per month to keep the ${lm://Field/1} data privat... = I would pay £20 to keep the data private

CondA_MoGPS_10
${lm://Field/1}

Would you be willing to pay £10 per month to keep the **${lm://Field/1}** data private?

- I would pay £10 to keep the data private (1)
- I would not pay £10 to keep the data private (2)

Skip To: End of Block If ${lm://Field/1} Would you be willing to pay £10 per month to keep the ${lm://Field/1} data private? = I would pay £10 to keep the data private

Skip To: End of Block If ${lm://Field/1} Would you be willing to pay £10 per month to keep the ${lm://Field/1} data private? = I would not pay £10 to keep the data private

CondA_MoGPS_1pound
${lm://Field/1}

Would you be willing to pay £1 per month to keep the **${lm://Field/1}** data private?

- I would pay £1 to keep the data private (1)
- I would not pay £1 to keep the data private (2)

Skip To: End of Block If ${lm://Field/1} Would you be willing to pay £1 per month to keep the ${lm://Field/1} data private... = I would pay £1 to keep the data private

Skip To: CondA_MoGPS_1penny If ${lm://Field/1} Would you be willing to pay £1 per month to keep the ${lm://Field/1} data private... = I would not pay £1 to keep the data private

CondA_MoGPS_1penny
${lm://Field/1}

Would you be willing to pay 1p per month to keep the **${lm://Field/1}** data private?

- I would pay 1p to keep the data private (1)
- I would not pay 1p to keep the data private (2)

Skip To: End of Block If ${lm://Field/1} Would you be willing to pay 1p per month to keep the ${lm://Field/1} data private?  = I would pay 1p to keep the data private

Skip To: End of Block If ${lm://Field/1} Would you be willing to pay 1p per month to keep the ${lm://Field/1} data private?  = I would not pay 1p to keep the data private

CondA_MoGPS_Max
${lm://Field/1}

You said you would be willing to pay £200 per month to keep the **${lm://Field/1}** data private. What is the maximum amount of money you would be willing to pay per month to keep this data private?

- Each month, I would pay up to £ (1) ________________________________________________

End of Block: CondA_Mobile GPS

Start of Block: CondA_Banking

CondA_BANK_intro **${lm://Field/1}**
When you first use the personal data app you are given two choices; either you can run the app in a “free” mode or or you can use it in “paid” mode. In “free” mode, you do not have to pay anything but the company providing the app will collect information about you when you use it. In “paid” mode, you pay money for the service and your data will remain private. If you choose this option, your data will not be used by the company providing the service for any purposes apart from maintaining the functionality of the app.

CondA_BANK_any Would you be willing to pay something, even a very small amount, to keep the **${lm://Field/1}** data private, or would you use the app for free and share the data with the app provider?

- I would pay something to keep the data private. (1)
- I would use the app for free and share the data. (2)

Skip To: CondA_BANK_40 If Would you be willing to pay something, even a very small amount, to keep the ${lm://Field/1} data... = I would pay something to keep the data private.

Skip To: End of Block If Would you be willing to pay something, even a very small amount, to keep the ${lm://Field/1} data... = I would use the app for free and share the data.

CondA_BANK_40
${lm://Field/1}

Would you be willing to pay £40 per month to keep the **${lm://Field/1}** data private?

- I would pay £40 to keep the data private. (1)
- I would not pay £40 to keep the data private. (2)

Skip To: CondA_BANK_125 If ${lm://Field/1} Would you be willing to pay £40 per month to keep the ${lm://Field/1} data privat... = I would pay £40 to keep the data private.

Skip To: CondA_BANK_15 If ${lm://Field/1} Would you be willing to pay £40 per month to keep the ${lm://Field/1} data privat... = I would not pay £40 to keep the data private.

CondA_BANK_125
${lm://Field/1}

Would you be willing to pay £125 per month to keep the **${lm://Field/1}** data private?

- I would pay £125 to keep the data private. (1)
- I would not pay £125 to keep the data private. (2)

Skip To: CondA_BANK_175 If ${lm://Field/1} Would you be willing to pay £125 per month to keep the ${lm://Field/1} data priva... = I would pay £125 to keep the data private.

Skip To: CondA_BANK_75 If ${lm://Field/1} Would you be willing to pay £125 per month to keep the ${lm://Field/1} data priva... = I would not pay £125 to keep the data private.

CondA_BANK_15
${lm://Field/1}

Would you be willing to pay £15 per month to keep the **${lm://Field/1}** data private?

- I would pay £15 to keep the data private. (1)
- I would not pay £15 to keep the data private. (2)

Skip To: CondA_BANK_25 If ${lm://Field/1} Would you be willing to pay £15 per month to keep the ${lm://Field/1} data privat... = I would pay £15 to keep the data private.

Skip To: CondA_BANK_5 If ${lm://Field/1} Would you be willing to pay £15 per month to keep the ${lm://Field/1} data privat... = I would not pay £15 to keep the data private.

CondA_BANK_175
${lm://Field/1}

Would you be willing to pay £175 per month to keep the **${lm://Field/1}** data private?

- I would pay £175 to keep the data private (1)
- I would not pay £175 to keep the data private (2)

Skip To: CondA_BANK_200 If ${lm://Field/1} Would you be willing to pay £175 per month to keep the ${lm://Field/1} data priva... = I would pay £175 to keep the data private

Skip To: CondA_BANK_150 If ${lm://Field/1} Would you be willing to pay £175 per month to keep the ${lm://Field/1} data priva... = I would not pay £175 to keep the data private

CondA_BANK_75
${lm://Field/1}

Would you be willing to pay £75 per month to keep the **${lm://Field/1}** data private?

- I would pay £75 to keep the data private (1)
- I would not pay £75 to keep the data private (2)

Skip To: CondA_BANK_100 If ${lm://Field/1} Would you be willing to pay £75 per month to keep the ${lm://Field/1} data privat... = I would pay £75 to keep the data private

Skip To: CondA_BANK_50 If ${lm://Field/1} Would you be willing to pay £75 per month to keep the ${lm://Field/1} data privat... = I would not pay £75 to keep the data private

CondA_BANK_25
${lm://Field/1}

Would you be willing to pay £25 per month to keep the **${lm://Field/1}** data private?

- I would pay £25 to keep the data private (1)
- I would not pay £25 to keep the data private (2)

Skip To: CondA_BANK_30 If ${lm://Field/1} Would you be willing to pay £25 per month to keep the ${lm://Field/1} data privat... = I would pay £25 to keep the data private

Skip To: CondA_BANK_20 If ${lm://Field/1} Would you be willing to pay £25 per month to keep the ${lm://Field/1} data privat... = I would not pay £25 to keep the data private

CondA_BANK_5
${lm://Field/1}

Would you be willing to pay £5 per month to keep the **${lm://Field/1}** data private?

- I would pay £5 to keep the data private (1)
- I would not pay £5 to keep the data private (2)

Skip To: CondA_BANK_10 If ${lm://Field/1} Would you be willing to pay £5 per month to keep the ${lm://Field/1} data private... = I would pay £5 to keep the data private

Skip To: CondA_BANK_1pound If ${lm://Field/1} Would you be willing to pay £5 per month to keep the ${lm://Field/1} data private... = I would not pay £5 to keep the data private

CondA_BANK_200
${lm://Field/1}

Would you be willing to pay £200 per month to keep the **${lm://Field/1}** data private?

- I would pay £200 to keep the data private (1)
- I would not pay £200 to keep the data private (2)

Skip To: CondA_BANK_max If ${lm://Field/1} Would you be willing to pay £200 per month to keep the ${lm://Field/1} data priva... = I would pay £200 to keep the data private

Skip To: End of Block If ${lm://Field/1} Would you be willing to pay £200 per month to keep the ${lm://Field/1} data priva... = I would not pay £200 to keep the data private

CondA_BANK_150
${lm://Field/1}

Would you be willing to pay £150 per month to keep the **${lm://Field/1}** data private?

- I would pay £150 to keep the data private (1)
- I would not pay £150 to keep the data private (2)

Skip To: End of Block If ${lm://Field/1} Would you be willing to pay £150 per month to keep the ${lm://Field/1} data priva... = I would pay £150 to keep the data private

Skip To: End of Block If ${lm://Field/1} Would you be willing to pay £150 per month to keep the ${lm://Field/1} data priva... = I would not pay £150 to keep the data private

CondA_BANK_100
${lm://Field/1}

Would you be willing to pay £100 per month to keep the **${lm://Field/1}** data private?

- I would pay £100 to keep the data private (1)
- I would not pay £100 to keep the data private (2)

Skip To: End of Block If ${lm://Field/1} Would you be willing to pay £100 per month to keep the ${lm://Field/1} data priva... = I would pay £100 to keep the data private

Skip To: End of Block If ${lm://Field/1} Would you be willing to pay £100 per month to keep the ${lm://Field/1} data priva... = I would not pay £100 to keep the data private

CondA_BANK_50
${lm://Field/1}

Would you be willing to pay £50 per month to keep the **${lm://Field/1}** data private?

- I would pay £50 to keep the data private (1)
- I would not pay £50 to keep the data private (2)

Skip To: End of Block If ${lm://Field/1} Would you be willing to pay £50 per month to keep the ${lm://Field/1} data privat... = I would pay £50 to keep the data private

Skip To: End of Block If ${lm://Field/1} Would you be willing to pay £50 per month to keep the ${lm://Field/1} data privat... = I would not pay £50 to keep the data private

CondA_BANK_30
${lm://Field/1}

Would you be willing to pay £30 per month to keep the **${lm://Field/1}** data private?

- I would pay £30 to keep the data private (1)
- I would not pay £30 to keep the data private (2)

Skip To: End of Block If ${lm://Field/1} Would you be willing to pay £30 per month to keep the ${lm://Field/1} data privat... = I would pay £30 to keep the data private

Skip To: End of Block If ${lm://Field/1} Would you be willing to pay £30 per month to keep the ${lm://Field/1} data privat... = I would not pay £30 to keep the data private

CondA_BANK_20
${lm://Field/1}

Would you be willing to pay £20 per month to keep the **${lm://Field/1}** data private, or would you use the app for free and share the data?

- I would pay £20 to keep the data private (1)
- I would not pay £20 to keep the data private (2)

Skip To: End of Block If ${lm://Field/1} Would you be willing to pay £20 per month to keep the ${lm://Field/1} data privat... = I would pay £20 to keep the data private

Skip To: End of Block If ${lm://Field/1} Would you be willing to pay £20 per month to keep the ${lm://Field/1} data privat... = I would pay £20 to keep the data private

CondA_BANK_10
${lm://Field/1}

Would you be willing to pay £10 per month to keep the **${lm://Field/1}** data private?

- I would pay £10 to keep the data private (1)
- I would not pay £10 to keep the data private (2)

Skip To: End of Block If ${lm://Field/1} Would you be willing to pay £10 per month to keep the ${lm://Field/1} data private? = I would pay £10 to keep the data private

Skip To: End of Block If ${lm://Field/1} Would you be willing to pay £10 per month to keep the ${lm://Field/1} data private? = I would not pay £10 to keep the data private

CondA_BANK_1pound
${lm://Field/1}

Would you be willing to pay £1 per month to keep the **${lm://Field/1}** data private?

- I would pay £1 to keep the data private (1)
- I would not pay £1 to keep the data private (2)

Skip To: End of Block If ${lm://Field/1} Would you be willing to pay £1 per month to keep the ${lm://Field/1} data private... = I would pay £1 to keep the data private

Skip To: CondA_BANK_1penny If ${lm://Field/1} Would you be willing to pay £1 per month to keep the ${lm://Field/1} data private... = I would not pay £1 to keep the data private

CondA_BANK_1penny
${lm://Field/1}

Would you be willing to pay 1p per month to keep the **${lm://Field/1}** data private?

- I would pay 1p to keep the data private (1)
- I would not pay 1p to keep the data private (2)

Skip To: End of Block If ${lm://Field/1} Would you be willing to pay 1p per month to keep the ${lm://Field/1} data private?  = I would pay 1p to keep the data private

Skip To: End of Block If ${lm://Field/1} Would you be willing to pay 1p per month to keep the ${lm://Field/1} data private?  = I would not pay 1p to keep the data private

CondA_BANK_max
${lm://Field/1}

You said you would be willing to pay £200 per month to keep the **${lm://Field/1}** data private. What is the maximum amount of money you would be willing to pay per month to keep this data private?

- Each month, I would pay up to £ (1) ________________________________________________

End of Block: CondA_Banking

Start of Block: Comments A

commentsA If you have any comments, please use the box below to add them.

________________________________________________________________

________________________________________________________________

________________________________________________________________

________________________________________________________________

________________________________________________________________

End of Block: Comments A

Start of Block: Condition B (L&M version)

| 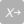 |
| --- |

Importance How important is it for you to keep ${lm://Field/1} data private as opposed to sharing it with the app provider? Please indicate on the scale from “**Not At All Important**” to “**Extremely Important**”.

- Not At All Important (1)
- Slightly Important (2)
- Moderately Important (3)
- Very Important (4)
- Extremely Important (5)

End of Block: Condition B (L&M version)

Start of Block: Comments B

commentsB If you have any comments, please use the box below to add them.

________________________________________________________________

________________________________________________________________

________________________________________________________________

________________________________________________________________

________________________________________________________________

End of Block: Comments B

Start of Block: Condition C

SliderIntro When you first use the personal data app you are given two choices; either you can run the app in a “free” mode or or you can use it in “paid” mode. In “free” mode, you do not have to pay anything but the company providing the app will collect information about you when you use it. In “paid” mode, you pay money for the service and your data will remain private. If you choose this option, your data will not be used by the company providing the service for any purposes apart from maintaining the functionality of the app.

| 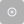 |
| --- |

Slider
Please use the slider to assign the largest amount of money you would be willing to pay per month to use the app in paid mode, keeping ${lm://Field/1} data private. If you would prefer to use the app in the "free" mode set the slider on 0. 


 
(The amounts are in GBP)

|  | 0 | 25 | 50 | 75 | 100 | 125 | 150 | 175 | 200 |
| --- | --- | --- | --- | --- | --- | --- | --- | --- | --- |

| () | 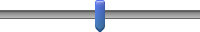 |
| --- | --- |

End of Block: Condition C

Start of Block: Comments C

commentsC If you have any comments, please use the box below to add them.

________________________________________________________________

________________________________________________________________

________________________________________________________________

________________________________________________________________

________________________________________________________________

End of Block: Comments C

Start of Block: Condition D

| 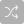 | 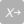 |
| --- | --- |

Ranking Using the app requires you to share some pieces of your data with the app providers. All the pieces of personal data are listed below. We would like to know how you would rank them.

 The **top** of the ranking should be the data you would be **the most willing to share** with the app provider, if you needed to use the service for free. The **bottom** of the ranking should be the data you would be **the most unwilling to share** with the app provider.

 Drag and drop the different pieces of information to create your ranking

______ Banking Transactions data (1)

______ Browsing/Search/Click History data (2)

______ Electricity Use at Home data (3)

______ Loyalty Cards data (4)

______ Medical Records data (5)

______ Mobile phone GPS data (6)

______ Physical Activity (Exercise) Tracking data (7)

______ Social Media data (8)

CommentsD If you have any comments, please use the box below to add them.

________________________________________________________________

________________________________________________________________

________________________________________________________________

________________________________________________________________

________________________________________________________________

End of Block: Condition D

Start of Block: Condition E

| 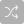 | 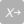 |
| --- | --- |

Pairwise Using the app requires you to share some pieces of your data with the app provider. Here are two pieces of personal data: **${lm://Field/1}** and **${lm://Field/2}**. Which would you rather give away if you needed to use the service for free?

- Give away **${lm://Field/1}** (1)
- Give away **${lm://Field/2}** (2)

End of Block: Condition E

Start of Block: Comments E

commentsE If you have any comments, please use the box below to add them.

________________________________________________________________

________________________________________________________________

________________________________________________________________

________________________________________________________________

________________________________________________________________

End of Block: Comments E

| Page Break |  |
| --- | --- |

Comments Thank  you for your answers. If you have any comments about the survey, please enter them below. 


Don't forget to click the ">>" button to proceed to Prolific and claim your reward.

________________________________________________________________

________________________________________________________________

________________________________________________________________

________________________________________________________________

________________________________________________________________

End of Block: Good bye
