# Supplementary material for: Unpacking privacy: Valuation of personal data protection
Source: PLoS One. 2023 May 3;18(5):e0284581. doi: 10.1371/journal.pone.0284581 (PMC10156004; doi:10.1371/journal.pone.0284581)
Supplement: Attachment — Submitted filename: PONE-Report.pdf [file pone.0284581.s006.pdf]

PONE-D-22-24992-R1

The authors have followed my suggestion and acknowledged that they are not prescribing a market where consumers pay to protect their data privacy in the introduction section (on page 10 of the revised manuscript in lines 232-240) and in the discussion section (on page 39 of the revised manuscript in lines 776-786). The authors have also attempted to use the word valuation rather than willingness to pay to correctly reflect the purpose of the study in most places. However, the exposition of the paper still falls short of emphasizing that point in some vital places. Here are the suggestions to rectify that problem.

1. Title: “Unpacking Privacy: Willingness to Pay to Protect Personal Data” is the present title of the paper. The title itself is prone to be interpreted as propagating a market to pay for personal data protection. An alternative rephrasing like the following could solve the issue: “Unpacking Privacy: Valuation of Personal Data Protection.”

2. Abstract: The following sentence in the abstract also needs further clarification or change. *“Further, they might be willing to pay for an otherwise free service if paying allowed them to avoid sharing personal data.”* One can think of several ways to clarify that the willingness to pay is just a tool that the authors are using to elicit consumer valuation of data privacy, and the paper is not recommending the creation of a market where consumers pay for data privacy as there are ethical concerns involved.

If these are corrected, I am willing to recommend accepting the paper.
